# Optimizing Biophysical Large-Scale Brain Circuit Models With Deep Neural Networks

**DOI:** 10.1101/2025.04.07.647497

**Published:** 2025-04-07

**Authors:** Tianchu Zeng, Fang Tian, Shaoshi Zhang, Xin Li, Ai Peng Tan, Bart Larsen, Ruben C. Gur, Raquel E. Gur, Tyler M. Moore, Theodore D. Satterthwaite, Gustavo Deco, Avram J. Holmes, B.T. Thomas Yeo

**Affiliations:** 1 Centre for Sleep & Cognition & Centre for Translational Magnetic Resonance Research, Yong Loo Lin School of Medicine, National University of Singapore, Singapore; 2 Department of Electrical and Computer Engineering, National University of Singapore, Singapore; 3 N.1 Institute for Health, National University of Singapore, Singapore; 4 Department of Medicine, Heathy Longevity Translational Research Programme, Human Potential Translational Research Programme & Institute for Digital Medicine (WisDM), Yong Loo Lin School of Medicine, National University of Singapore, Singapore; 5 Integrative Sciences and Engineering Programme (ISEP), National University of Singapore, Singapore; 6 Singapore Institute for Clinical Sciences (SICS), Agency for Science, Technology and Research (A*STAR), Singapore; 7 Department of Diagnostic Radiology, Yong Loo Lin School of Medicine, National University of Singapore, Singapore; 8 Department of Pediatrics, University of Minnesota, MN, USA; 9 Masonic Institute for the Developing Brain, University of Minnesota, Minneapolis, MN, United States; 10 Department of Psychiatry, University of Pennsylvania, Philadelphia, PA, USA; 11 Lifespan Brain Institute (LiBI) of Penn Medicine and CHOP, University of Pennsylvania, Philadelphia, PA, USA; 12 Department of Radiology, University of Pennsylvania, Philadelphia, PA, USA; 13 Penn Lifespan Informatics and Neuroimaging Center, University of Pennsylvania, Philadelphia, PA, USA; 14 Center for Brain and Cognition, Department of Technology and Information, Universitat Pompeu Fabra, Barcelona, Spain; 15 Institució Catalana de la Recerca i Estudis Avançats, Universitat Barcelona, Barcelona, Spain; 16 Department of Psychiatry, Brain Health Institute, Rutgers University, Piscataway, NJ, United States; 17 Center for Advanced Human Brain Imaging Research, Rutgers University, Piscataway, NJ, United States; 18 Athinoula A. Martinos Center for Biomedical Imaging, Massachusetts General Hospital, Charlestown, USA

## Abstract

Biophysical modeling provides mechanistic insights into brain function, from single-neuron dynamics to large-scale circuit models bridging macro-scale brain activity with microscale measurements. Biophysical models are governed by biologically meaningful parameters, many of which can be experimentally measured. Some parameters are unknown, and optimizing their values can dramatically improve adherence to experimental data, significantly enhancing biological plausibility. Previous optimization methods – such as exhaustive search, gradient descent, evolutionary strategies and Bayesian optimization – require repeated, computationally expensive numerical integration of biophysical differential equations, limiting scalability to population-level datasets. Here, we introduce DELSSOME (DEep Learning for Surrogate Statistics Optimization in MEan field modeling), a framework that bypasses numerical integration by directly predicting whether model parameters produce realistic brain dynamics. When applied to the widely used feedback inhibition control (FIC) mean field model, DELSSOME achieves a 2000× speedup over Euler integration. By embedding DELSSOME within an evolutionary optimization strategy, trained models generalize to new datasets without additional tuning, enabling a 50× speedup in FIC model estimation while preserving neurobiological insights. The massive acceleration facilitates large-scale mechanistic modeling in population-level neuroscience, unlocking new opportunities for understanding brain function.

## 1 Introduction

Biophysical modeling is a powerful approach for deriving mechanistic principles of brain function. The Nobel Prize-winning Hodgkin–Huxley model exemplifies this approach by mathematically explaining action potential generation in single neurons (Hodgkin & Huxley, 1952). As another example, mean field models (MFMs) have yielded biophysically-plausible accounts of perceptual decision making (Brunel & Wang, 2001; Wong & Wang, 2006), while large-scale circuit models of coupled MFMs have revealed anatomical scaffolding of global spontaneous brain activity (Honey et al., 2009; Breakspear et al., 2010; Deco et al., 2014; Hansen et al., 2015). Large-scale circuit models have also bridged macro-scale brain dynamics with microscale measurements, including histological cell density (Wang et al., 2019) and transcriptomic data (Deco et al., 2021).

The dynamical properties of biophysical models are governed by biologically meaningful parameters (e.g., membrane resting potentials), many of which can be determined from cellular neurophysiology or histology. However, some parameters are unknown and require tuning. The tuning process usually involves iteratively integrating biophysical differential equations and adjusting parameter values to maximize similarity between simulated and empirical brain activity, such as resting-state functional MRI (rs-fMRI). The most common approach for optimizing model fit is an exhaustive parameter search (Kringelbach et al., 2020; Müller et al., 2023; Pang et al., 2023). However, the search space grows exponentially with the number of parameters, so most studies optimize only a single parameter (Deco et al., 2014; Müller et al., 2023; Pang et al., 2023).

To optimize a larger number of parameters, gradient descent with approximate gradients (Wang et al., 2019) or exact gradients (Deistler et al., 2024) have been proposed. However, exact gradients require the cost function to be differentiable. In contrast, evolutionary and Bayesian optimization methods iteratively sample candidate parameters from a proposal distribution, which is then updated based on their goodness of fit (Demirtaş et al., 2019; Kong et al., 2021). For example, most large-scale circuit models assume uniform local synaptic properties (Freyer et al., 2012; Deco et al., 2014; Trakoshis et al., 2020), despite well-documented spatial heterogeneity in cortical architecture (Kim et al., 2017; Burt et al., 2018; Froudist-Walsh et al., 2021; Zhang et al., 2024b). Using a Bayesian (Demirtaş et al., 2019) or evolutionary (Kong et al., 2021) strategy to optimize spatially heterogeneous synaptic parameters to rs-fMRI results in markedly more realistic brain dynamics. The fitted large-scale circuit models can also generate a marker of whole-cortex excitation/inhibition (E/I) ratio, which has been validated with pharmacological fMRI and shows strong spatial convergence with positron emission tomography (Zhang et al., 2024a).

A key limitation of previous approaches is the need to numerically integrate differential equations during optimization, which is computationally expensive. Emerging deep neural networks (DNNs) in other domains seek to learn the mapping between differential equation parameters and the resulting simulated (numerically integrated) time courses (Raissi et al., 2019; Li et al., 2021; Kovachki et al., 2023). A feedforward pass through a DNN is extremely fast, so a trained DNN can bypass computationally expensive numerical integration. Since numerical integration can generate unlimited training data, this problem is especially well-suited for data-hungry DNNs. However, DNN-based methods focus on deterministic differential equations and replace at most hundreds of numerical integration steps (Li et al., 2021; Kovachki et al., 2023). In contrast, biophysical brain models rely on stochastic differential equations and require over 100,000 integration steps for tens or hundreds of brain regions (Lam et al., 2022; Pang et al., 2023; Zhang et al., 2024a). Naively applying existing DNN methods would require predicting millions of variables, which is both challenging and computationally expensive.

Here, we propose DELSSOME (DEep Learning for Surrogate Statistics Optimization in MEan field modeling), a framework for optimizing large-scale circuit models involving stochastic differential equations. Instead of predicting entire time courses like previous DNN approaches, DELSSOME directly predicts whether a given parameter set produces realistic brain dynamics based on a rich set of neuroscience-informed surrogate statistics, thus reducing the prediction targets from millions of variables to several key metrics. We apply DELSSOME to the widely used feedback inhibition control (FIC) mean field model (Deco et al., 2014) using data from the Human Connectome Project (HCP; Glasser et al., 2013; Van Essen et al., 2013). DELSSOME accurately predicts FIC model realism, achieving a 2000× speedup over Euler integration. When embedded within an evolutionary optimization strategy, DELSSOME estimates high quality FIC model parameters, validated against Euler integration. DELSSOME models trained on the HCP generalize to the Philadelphia Neurodevelopmental Cohort (PNC) without any further tuning, offering a 50× speedup over Euler integration, while preserving neurodevelopmental insights. The DELSSOME framework can be in principle applied to other biophysical brain models and even differential equations from other domains. The massive acceleration provided by DELSSOME enables the deployment of biophysical models in population-level datasets, paving the way for new mechanistic insights into the brain.

## 2 Results

### 2.1 Optimizing the feedback inhibition control (FIC) model with numerical integration

**Figure 1.**
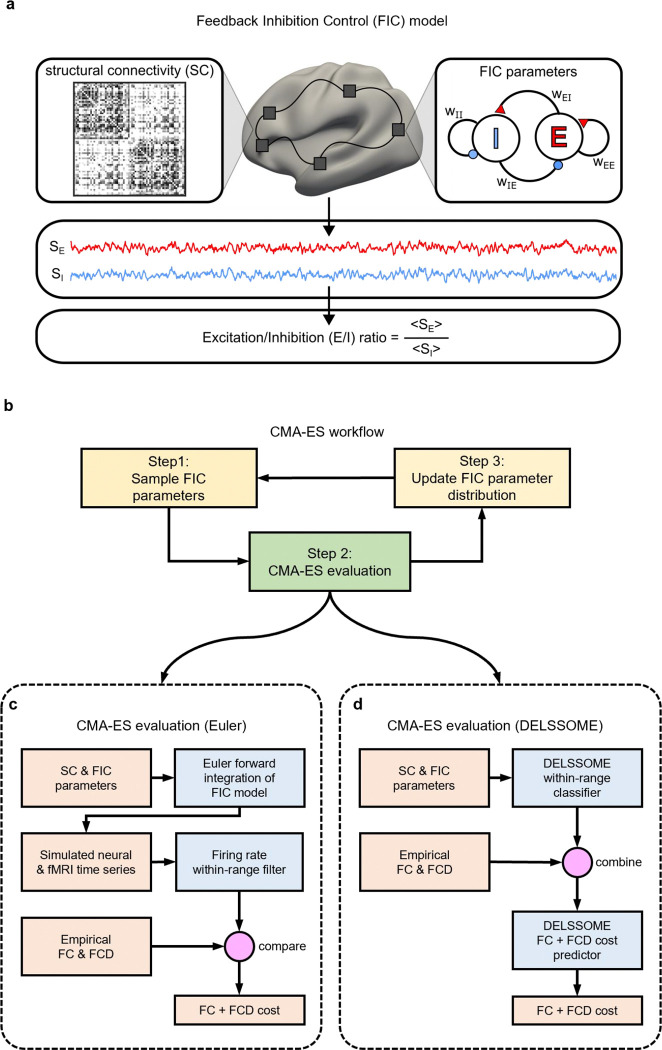
Overview of the Feedback Inhibition Control (FIC) model and its optimization with the covariance matrix adaptation evolution strategy (CMA-ES). **a.** The FIC model (Deco et al., 2014) comprises differential equations describing the neural dynamics of excitatory (“E”) and inhibitory (“I”) neuronal populations within each cortical region (right panel). Excitatory connections are represented by red triangles, and inhibitory connections are represented by blue circles. Connection strengths between neuronal populations are denoted as “w_XY_ ”, where x is the source population and y is the target population (e.g., w_EI_ represents the connection strength from the excitatory to the inhibitory population). Cortical regions are interconnected via excitatory connections parameterized by a structural connectivity (SC) matrix. For a given set of model parameters, time courses of excitatory (S_E_ ) and inhibitory (S_I_) synaptic gating variables, which represent the fraction of open channels, can be simulated. The excitation-inhibition ratio (E/I ratio) is defined as the ratio of the temporal average of S_E_ to S_I_ (Zhang et al., 2024a). **b.** 100 sets of FIC parameters are sampled from an initial Gaussian distribution (Step 1). The 100 sets of FIC parameters are then evaluated (Step 2). The 10 sets of FIC parameters with the best evaluation metric are then used to update the Gaussian distribution (Step 3). **c.** To evaluate a set of FIC parameters, the FIC model is numerically integrated resulting in simulated neural and fMRI time courses. FIC parameters with simulated excitatory firing rate outside the physiologically plausible range of 2.7 to 3.3 Hz (De Kock & Sakmann, 2008) are considered out of range and removed from further consideration. Simulated fMRI time courses (with valid neural excitatory firing rate) are then evaluated by computing a cost function that compares simulated and empirical functional connectivity (FC), as well as simulated and empirical functional connectivity dynamics (FCD), which we will refer to as FC+FCD cost. A lower FC+FCD cost indicates more realistic simulated fMRI time courses. **d.** Without performing numerical integration, the DELSSOME (Deep Learning for Surrogate Statistics Optimization in Mean Field Modeling) within-range classifier directly predicts whether a set of FIC parameters will lead to excitatory firing rates within the range of 2.7 to 3.3 Hz. For FIC parameters that survive the DELSSOME within-range classifier, the DELSSOME cost predictor will predict the FC+FCD cost without numerical integration.

The FIC model (Deco et al., 2014) is a neural mass model derived through a mean-field reduction of a spiking neuronal network model (Brunel & Wang, 2001; Wong & Wang, 2006). The model comprises ordinary differential equations (ODEs) that capture the dynamics of excitatory and inhibitory neuronal populations within each cortical region (Figure 1a; Supplementary Methods S1). Local dynamics are driven by recurrent interactions within the excitatory and inhibitory populations, as well as between excitatory and inhibitory populations. Higher excitatory-to-excitatory recurrent strength (w_EE_) and lower inhibitory-to-excitatory connection strength (w_IE_) increases activity of the excitatory population. Similarly, higher excitatory-to-inhibitory connection strength (w_EI_) and lower inhibitory-to-inhibitory recurrent strength (w_II_) enhance synaptic currents in the inhibitory population. The noise amplitude (σ) controls neuronal noise in each cortical region. Finally, the excitatory populations in different cortical regions are connected via a structural connectivity (SC) matrix, modulated by a global coupling constant G.

In our previous study (Zhang et al., 2024a), a FIC model with spatially heterogeneous excitatory-to-excitatory recurrent strength (w_EE_), excitatory-to-inhibitory connection strength (w_EI_) and noise amplitude (σ) was fitted to empirical fMRI data using the covariance matrix adaptation evolution strategy (CMA-ES; Hansen, 2006). There were 10 unknown parameters (see Supplementary Methods S2 for details). The optimized FIC model was then used to generate excitatory and inhibitory synaptic gating variable time courses S_E_ and S_I_. The E/I ratio estimate was defined as the ratio of the temporal average of S_E_ and S_I_ (Figure 1a).

To optimize the FIC model, CMA-ES samples 100 sets of candidate parameters from a randomly initialized 10-D Gaussian distribution (Step 1 in Figure 1b). The 10-D Gaussian corresponds to the fact that we are trying to optimize 10 parameters. Each set of candidate parameters is then used to compute an evaluation metric that measures the realism of the resulting FIC model (Step 2 in Figure 1b). The 10 sets of candidate parameters with the best evaluation metric are then used to update the sampling distribution for the next epoch (Step 3 in Figure 1b). These three steps constitute one epoch of the CMA-ES algorithm.

Each CMA-ES epoch involves 100 children, so with 100 epochs, we need to evaluate 10,000 sets of FIC parameters (Step 2 in Figure 1b), which is the most computationally expensive step of CMA-ES. More specifically, for a given set of FIC parameters, neural and fMRI timecourses are simulated via numerical (Euler) integration of the FIC differential equations (Figure 1c). Children parameters leading to simulated excitatory firing rates outside the physiologically plausible range of 2.7 to 3.3 Hz (De Kock & Sakmann, 2008) are considered out of range and removed from further consideration (Figure 1c).

The simulated fMRI time courses of the remaining children (whose firing rates were within the range of 2.7 to 3.3Hz) were then evaluated by comparison with empirical (real) fMRI time courses using three evaluation metrics (Figure 1c). First, the simulated and empirical time courses can be used to compute simulated and empirical functional connectivity (FC) matrices respectively. Each FC element reflects the synchronicity of the time courses of a given pair of brain regions. The similarity of the static and empirical FC was computed based on the Pearson’s correlation (*r*) between static and empirical FC. Because *r* does not account for differences in scale, we also computed the absolute difference (*d*) between the means of the empirical and simulated FC matrices (Demirtaş et al., 2019), where smaller *d* indicates greater similarity. The inclusion of *d* was necessary to prevent overly synchronized fMRI signals (Zhang et al., 2024a).

Finally, the simulated and empirical fMRI time courses were also used to compute simulated and empirical functional connectivity dynamics (FCD) matrices respectively. While FC measures the overall synchronicity of a pair of brain regions over the entire fMRI session, FCD measures how the synchronicity of a pair of brain regions fluctuates throughout the fMRI session. Dissimilarity between the FCD matrices was computed using the Kolmogorov–Smirnov (KS) distance (Hansen et al., 2015; Kong et al., 2021; Zhang et al., 2024a). The overall FC+FCD cost function was defined as *(1 − r) + d + KS*. A lower cost indicates greater realism in the simulated fMRI time courses. More details about CMA-ES are found in Supplementary Methods S2.

### 2.2 DELSSOME yielded 2000× speed up for evaluating FIC model realism

To avoid computationally intensive numerical integration (Figure 1c) in the CMA-ES evaluation, we proposed the DELSSOME (Deep Learning for Surrogate Statistics Optimization in Mean Field Modeling) within-range classifier to directly predict whether a set of FIC parameters will lead to within-range firing rates (Figure 1d). For FIC parameters that survived the DELSSOME within-range classifier, the DELSSOME FC+FCD cost predictor will predict the FC+FCD cost without numerical integration (Figure 1d). The DELSSOME neural network architectures and training procedures are detailed in Sections 4.2 and 4.3 respectively.

To evaluate whether DELSSOME can effectively predict FIC model realism, we divided 1029 Human Connectome Project (HCP) participants into training (N = 680), validation (N = 180) and test sets (N = 169). The training participants were “bootstrapped” to generate 640,000 training samples. The validation and test participants were similarly “bootstrapped” to generate 140,000 and 130,000 validation and test samples respectively. The training samples were used to train the DELSSOME within-range classifier and DELSSOME FC+FCD cost predictor, while the validation samples were used to tune the hyperparameters. The final trained DELSSOME within-range classifier and cost predictor were then applied to the 130,000 test samples. Details are found in Section 4.3.

**Figure 2.**
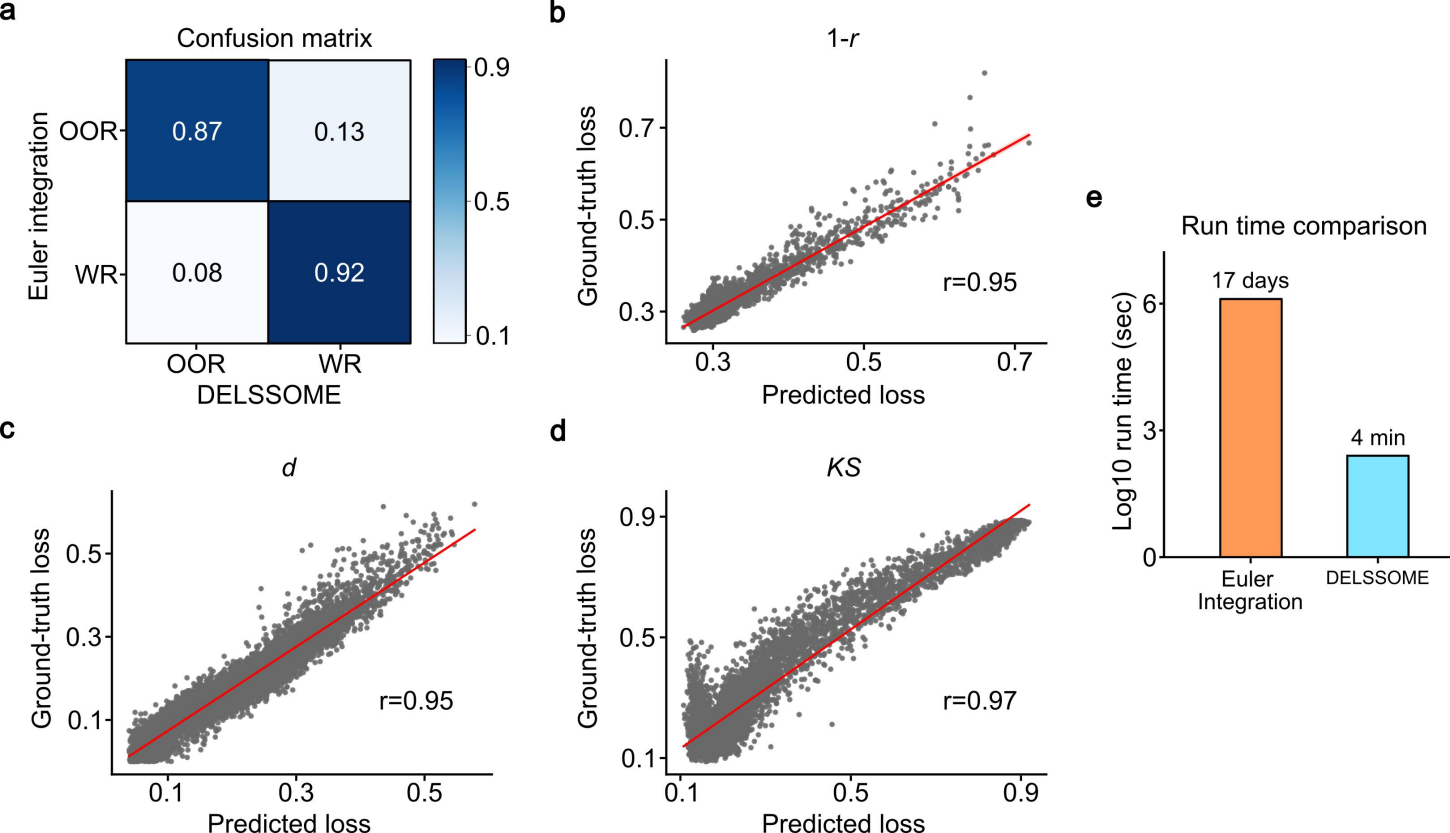
Test performance of DELSSOME within-range classifier and DELSSOME FC+FCD cost predictor. **a.** Test performance (confusion matrix) of DELSSOME within-range classifier. Overall accuracy was 90% (chance accuracy is 61%). OOR: out of range. WR: within range. **b.** Test performance of DELSSOME prediction of static FC cost (*1 - r*). **c.** Test performance of DELSSOME prediction of static FC cost (*d*). **d.** Test performance of DELSSOME prediction of FCD cost (*KS*). The correlation between the predicted and ground truth cost were at least 0.95. In all the analyses, ground truth was defined based on Euler integration, while the DELSSOME models avoided the Euler integration. **e.** Run time (log scale) of DELSSOME versus Euler integration in evaluating FIC model realism. DELSSOME offers a 2000× speed up over Euler integration.

In the HCP test set, 61% of FIC model parameters led to firing rates within the firing rate range of 2.7Hz to 3.3Hz (based on Euler integration). Therefore, a null classifier that simply predicted that all model parameters were within the firing rate would achieve an accuracy of 61%. In contrast, the DELSSOME within-range classifier achieved a classification accuracy of 90%, which was much higher than chance (Figure 2a). This suggests that the DELSSOME classifier can predict whether FIC parameters will lead to within-range firing rates without performing Euler integration.

The DELSSOME FC+FCD cost predictor also achieved high accuracy (r ≥ 0.95) when predicting the actual FC+FCD costs (obtained by Euler integration) in the HCP test set (Figures 2b to 2d). In particular, the DELSSOME cost predictor was able to predict the correlation cost (*1 - r*) between empirical and simulated static FC with r of 0.95 (Figure 2b), the absolute difference in mean between empirical and simulated static FC with r of 0.95 (Figure 2c), and the *KS* cost between empirical and simulated FCD with r of 0.97 (Figure 2d).

Figure 2 was obtained based on the 68-region Desikan-Killany cortical parcellation (Desikan et al., 2006). Similar performance (Figure S1) was obtained using a 100-region cortical parcellation (Yan et al., 2023). Overall, our results suggest that DELSSOME can predict whether the FIC model will generate realistic neural and fMRI dynamics without Euler integration. Because a single feedback pass through the DELSSOME deep neural network is extremely fast, DELSSOME offers a 2000× speed up over Euler integration for evaluating FIC model realism (Figures 2e & S1).

### 2.3 DELSSOME yielded 50× speed-up in the optimization of the FIC model

Although DELSSOME within-range classifier and DELSSOME cost predictor exhibited high accuracies (Figure 2), small discrepancies between the DELSSOME predictions and ground truth (obtained by Euler integration) might accumulate and compound if DELSSOME models were used to directly optimize the FIC model. Therefore, we tested whether DELSSOME models could replace Euler integration in the CMA-ES algorithm (Figure 1d).

**Figure 3.**
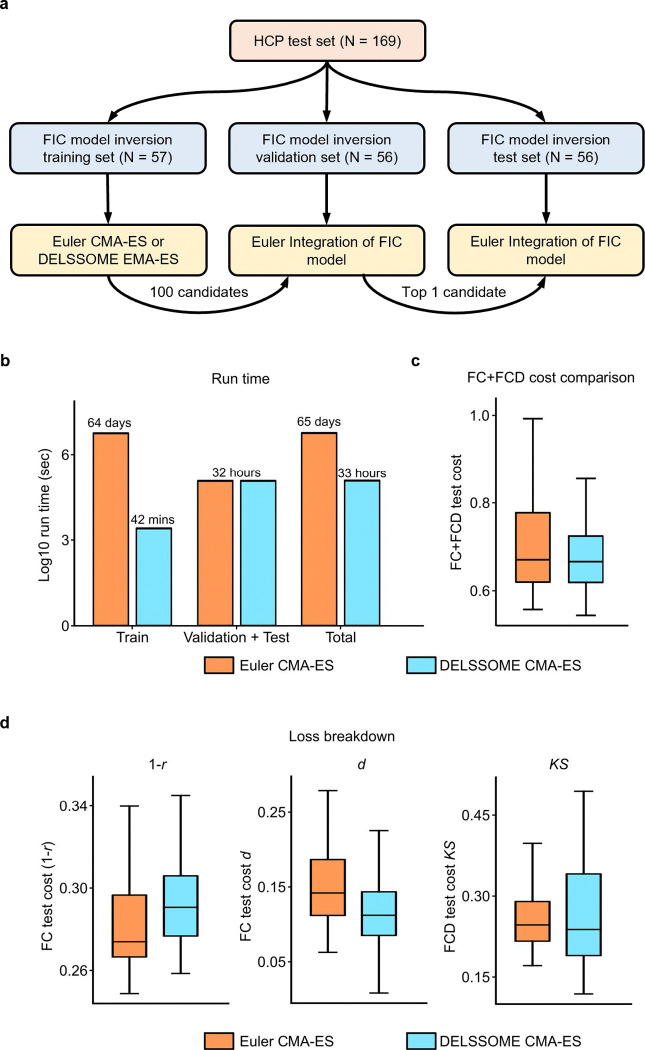
Comparison of Euler CMA-ES and DELSSOME CMA-ES in the HCP test set. **a.** Split of the HCP test set into FIC model inversion training, validation and test sets. Euler CMA-ES was run on the FIC model inversion training set and the 100 candidate parameter sets were evaluated in the FIC model inversion validation set. The best candidate parameter set was then evaluated in the test set. The procedure was repeated with DELSSOME CMA-ES replacing Euler CMA-ES in the training set. This comparison was repeated 50 times with different random initialization of the FIC model parameters each time. **b.** Run time (log scale) of DELSSOME CMA-ES versus Euler CMA-ES. DELSSOME CMA-ES offers a 2000× speed up over Euler CMA-ES in the training phase. If we also included validation and test phases in the run time, DELSSOME CMA-ES offers a 50× speed up over Euler CMA-ES. **c.** Total FC+FCD test cost comparison between DELSSOME CMA-ES and Euler CMA-ES. Each boxplot contains 50 data points corresponding to the 50 repetitions of the procedure in panel (a). **d.** Breakdown of the FC+FCD test cost from panel **c** into the two FC costs (*1-r* and *d*) and one FCD cost (*KS*). DELSSOME significantly sped up the estimation of the FIC model parameters without any degradation in estimation quality.

For this analysis, we only considered the HCP test participants (previous section) since they were not used to train the DELSSOME models. The HCP test participants were further divided into the FIC model inversion training set, validation set and test set (Figure 3a). Euler CMA-ES was run on the FIC model inversion training set for 100 epochs. The best candidate parameter set from each epoch was collated, yielding 100 candidate parameter sets. The 100 candidate parameter sets were then evaluated in the FIC model inversion validation set. Finally, the top parameter set from the validation set was evaluated in the FIC model inversion test set.

The same procedure was repeated with DELSSOME (replacing Euler integration) in the CMA-ES algorithm in the FIC model inversion training set, yielding 100 candidate parameter sets. These 100 candidate parameter sets were then evaluated in the FIC model inversion validation set based on Euler integration. The top parameter set from the validation set was then evaluated in the FIC model inversion test set, again based on Euler integration. More details can be found in Supplementary Methods S2.

During the training phase, DELSSOME CMA-ES was more than 2000 times faster than Euler CMA-ES (Figure 3b). However, during the validation and test phases, the run time was the same since both approaches utilize Euler integration (Figure 3b). Therefore, when we accounted for all training, validation and test phases, DELSSOME CMA-ES was around 50 times faster than Euler CMA-ES (Figure 3b).

On the other hand, FC+FCD costs between DELSSOME CMA-ES and Euler CMA-ES were similar, suggesting that the FIC parameters were equally good (Figure 3c). Figure 3 was obtained based on the 68-region Desikan-Killany cortical parcellation (Desikan et al., 2006). Similar performance (Figure S2) was obtained using a 100-region cortical parcellation (Yan et al., 2023). Overall, our results suggest that DELSSOME can greatly speed up the estimation of the FIC model parameters without any degradation in estimation quality.

### 2.4 DELSSOME generalizes to a new dataset without further tuning

To evaluate whether DELSSOME CMA-ES generalizes to a new dataset, and also whether DELSSOME CMA-ES can extract similar neurobiological information as Euler CMA-ES, we replicated key findings of our previous study showing that E/I ratio decreases with age during neurodevelopment (Zhang et al., 2024a). More specifically, the DELSSOME models trained from the HCP dataset (previous section) were applied directly to the Philadelphia Neurodevelopment Cohort (PNC) dataset (Satterthwaite et al., 2014; Calkins et al., 2015) without any further tuning.

**Figure 4.**
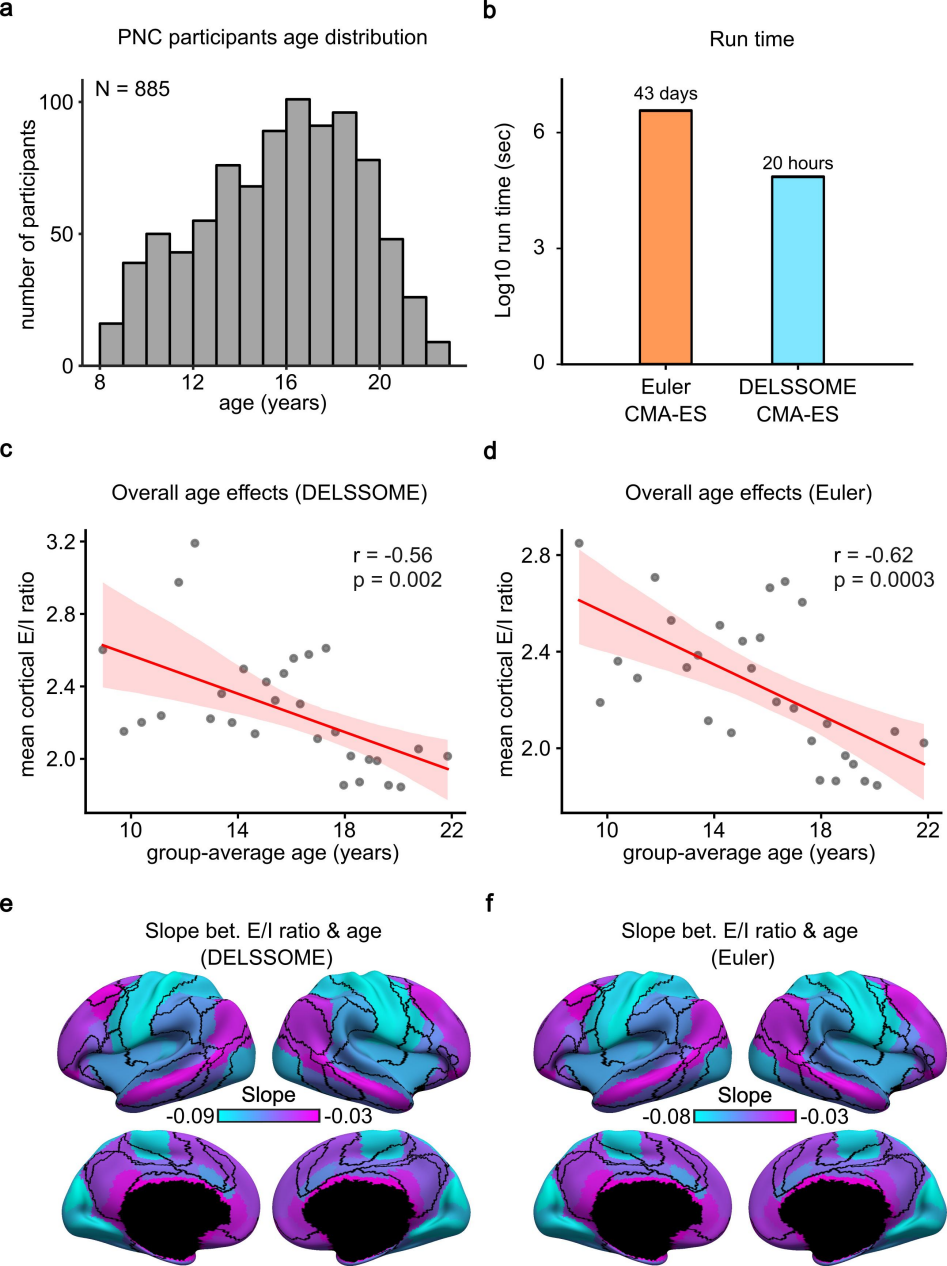
DELSSOME CMA-ES generalizes to the Philadelphia Neurodevelopmental Cohort (PNC) without further tuning. **a.** Age distribution of 885 PNC participants (mean = 15.66, std = 3.36, min = 8.17, max = 23). **b.** Run time comparison between DELSSOME CMA-ES and Euler CMA-ES. DELSSOME CMA-ES offers a 50× speed-up over Euler CMA-ES. **c.** Correlation between age and mean cortical E/I ratio estimated by DELSSOME CMA-ES. **d.** Correlation between age and mean cortical E/I ratio estimated by Euler CMA-ES. **e.** Regression slope between age and regional E/I ratio estimated by DELSSOME CMA-ES. **f.** Regression slope between age and regional E/I ratio estimated by Euler CMA-ES. All slopes in panels **e** and **f** are negative and significant after multiple comparisons correction with false discovery rate (FDR) q < 0.05.

The PNC dataset comprised 885 participants aged 8 to 23 years old (Figure 4a). Participants were sorted according to age (in months) and divided into 29 age groups (with 30 or 31 participants in each group). Within each age group, 15 participants were randomly selected as the validation set, while the remaining participants were assigned to the training set. For each age group, DELSSOME CMA-ES was applied to the training set for 50 epochs. The procedure was repeated five times with different random initializations, resulting in 250 candidate parameter sets. The 250 parameter sets were evaluated in the validation set with Euler integration. The best candidate parameter set was then used to generate an excitation/inhibition (E/I) ratio map using Euler integration. The same procedure was repeated with Euler CMA-ES. More details can be found in Supplementary Methods S5.

DELSSOME CMA-ES was around 50 times faster than Euler CMA-ES (Figure 4b). Consistent with the previous study (Zhang et al., 2024a), both DELSSOME CMA-ES and Euler EMA-ES revealed a decrease in mean cortical E/I ratio with age (Figures 4c and 4d). Pearson’s correlation between the 29 pairs of mean cortical E/I ratio (Figures 4c and 4d) was 0.88. The decrease in E/I ratio was also more pronounced in sensory-motor regions than association cortex for both DELSSOME CMA-ES and Euler CMA-ES (Figures 4e and 4f).

Since E/I ratio decreases with age (Figure 4), a lower E/I ratio might be an indicator of greater brain maturity. Therefore, another key result from our previous study was that among youth of the same age, lower E/I ratio was associated with better cognitive performance (Zhang et al., 2024a). Consequently, we repeated the same analysis as our previous study using DELSSOME CMA-ES trained from the HCP dataset.

**Figure 5.**
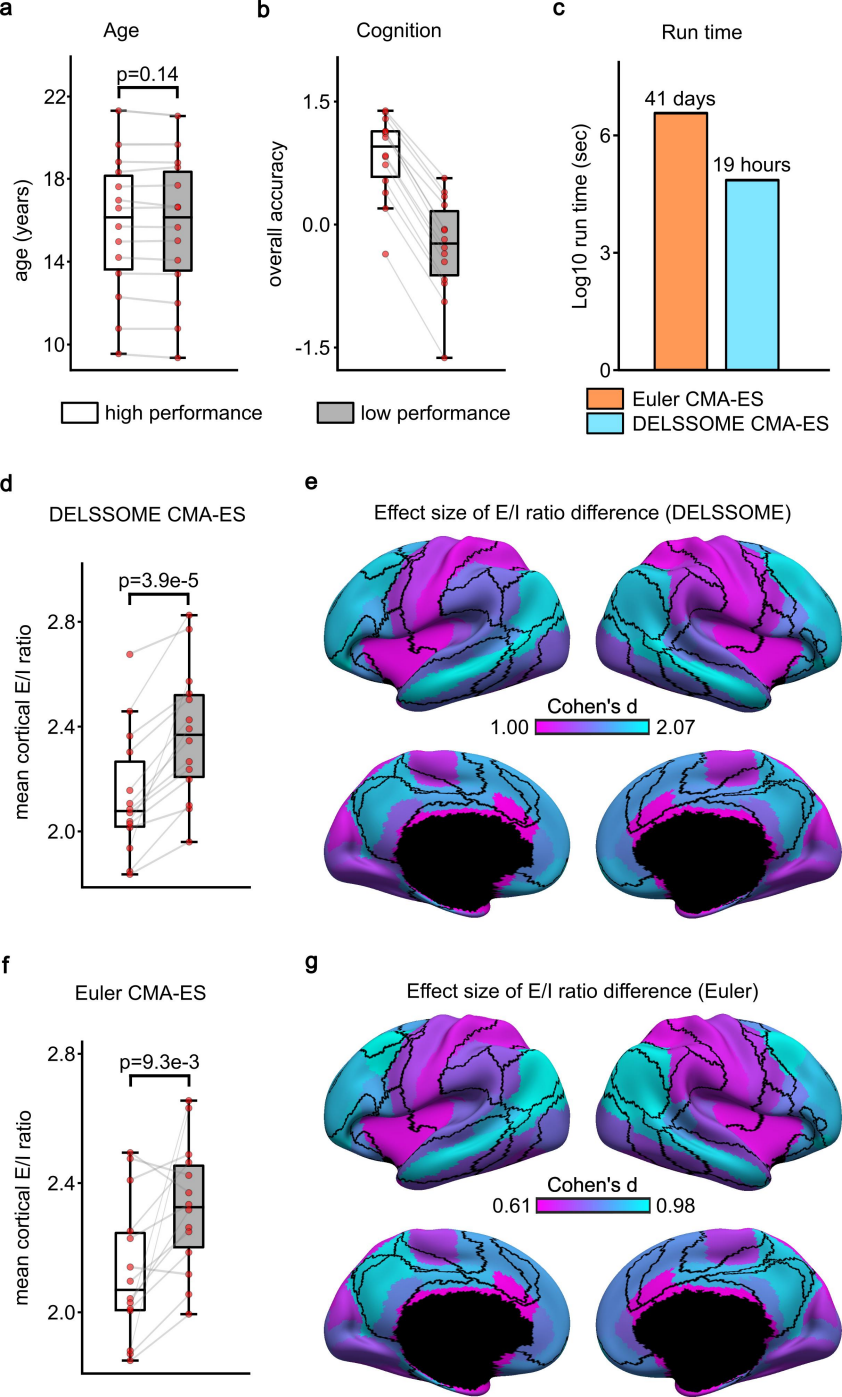
DELSSOME CMA-ES reveals lower E/I ratio in youth with high cognitive performance, consistent with Euler CMA-ES. **a.** Age of low and high cognitive performance groups. **b.** Cognitive performance (“overall accuracy”) of high and low performance groups. **c.** Run time comparison between DELSSOME CMA-ES and Euler CMA-ES. DELSSOME CMA-ES offers a 50× speed-up over Euler CMA-ES. **d.** Comparison of mean cortical E/I ratio between high-performance and low-performance groups estimated by DELSSOME CMA-ES. **e.** Regional differences in cortical E/I ratio between high-performance and low-performance groups estimated by DELSSOME CMA-ES. **f.** Comparison of mean cortical E/I ratio between high-performance and low-performance groups estimated by Euler CMA-ES. **g.** Regional differences in cortical E/I ratio between high-performance and low-performance groups estimated by Euler CMA-ES. All regional differences were significant after FDR correction with q < 0.05.

More specifically, we divided PNC participants into 14 high-performance and 14 low-performance groups. Each high-performance group was age-matched to a corresponding low-performance group (Figures 5a and 5b). Each low-performance or high-performance group comprised 31 or 32 participants. For each group, 15 participants were randomly assigned to the validation set, while the remaining participants were assigned to the training set. For each group, 250 candidate parameter sets were generated from the training set using DELSSOME CMA-ES. The 250 parameter sets were evaluated in the validation set with Euler integration. The best candidate parameter set was then used to generate an excitation/inhibition (E/I) ratio map using Euler integration. We then compared the E/I ratio between the high and low performance groups. The same procedure was repeated with Euler CMA-ES.

Similar to previous analyses, DELSSOME CMA-ES offered a 50 times speed up over Euler CMA-ES (Figure 5c). Both DELSSOME CMA-ES and Euler CMA-ES demonstrated that the age-matched high performance groups exhibited lower E/I ratio than the low performance groups (Figures 5d and 5f). Pearson’s correlation between the 28 pairs of E/I ratio (from Figures 5d and 5f) was 0.87. The association between E/I ratio and cognition was also more pronounced in association cortex than sensory-motor regions for both DELSSOME CMA-ES and Euler CMA-ES (Figures 5e and 5g).

The previous analyses (Figures 4 and 5) were performed using the 68-region Desikan-Killiany parcellation. Similar results were obtained with the 100-region Yan parcellation (Figures S3 and S4). Overall, these results underscore the robustness of the DELSSOME CMA-ES approach in replicating previous findings relating E/I ratio and cognitive development, suggesting that DELSSOME CMA-ES can be used to replace Euler CMA-ES in future neuroscientific studies.

## 3 Discussion

In this study, we proposed a deep learning framework, DELSSOME, which greatly accelerated the optimization of biophysical model parameters. In the case of the widely used FIC model, DELSSOME provided a 2000× speed up of the evaluation of the FIC model parameters, compared with Euler integration. When incorporated into an evolutionary optimization strategy, the resulting DELSSOME CMA-ES enjoyed a 50× speed up in the FIC model parameter estimation compared with Euler CMA-ES. DELSSOME trained in the HCP dataset also generalized to the PNC dataset without the need for further tuning. The speed up was achieved with no degradation in model quality (as measured by the FC-FCD cost evaluated using Euler integration), while generating equivalent neurodevelopment insights as our previous study (Zhang et al., 2024a).

### 3.1 Model architecture

In the case of the FIC model, DELSSOME comprised two DNNs: a within-range DNN classifier to predict whether firing rates are within-range and a DNN cost predictor to predict the FC+FCD cost. The two DNNs share similar structure. First, both DNNs utilize multiple layer perceptrons (MLPs) that take in SC and FIC parameters to generate a model embedding, which is simply a vector representing the intrinsic dynamics of the FIC model. In the case of the DELSSOME within-range classifier, the model embedding provides enough information to predict whether firing rates are within-range. In the case of the DELSSOME FC+FCD cost predictor, we are trying to predict the disagreement between simulated and empirical fMRI data, so we also need to generate an embedding of empirical FC and FCD. The empirical FC+FCD embedding can then be added with the model embedding to predict the FC+FCD cost.

This procedure of computing model and FC+FCD embeddings before adding them together parallels multimodal vision-language modeling approaches, such as CLIP (Contrastive Language-Image Pre-training; Radford et al., 2021), which employed a high-dimensional hidden space to align image and natural language embeddings. By leveraging a shared embedding space, we enabled direct comparison and integration of information between the SC and FIC parameters, as well as between SC+FIC parameters and empirical FC+FCD. Without the alignment provided by the shared embedding space, the different components (SC, FIC parameters, FC, FCD) are not comparable and cannot be integrated. Furthermore, the embeddings of the different components are added together, instead of concatenated, which is a strategy akin to integrating positional encoding and word encoding in the popular transformer architecture (Vaswani et al., 2017; Devlin et al., 2019).

### 3.2 Model performance

Despite the high accuracies of the DELSSOME within-range classifier and FC+FCD cost predictor (Figure 2), it was important to demonstrate that DELSSOME models were sufficiently accurate to optimize FIC model parameters (Figure 3). Indeed, early versions of the DELSSOME framework exhibited lower accuracy resulting in an accumulation of errors over CMA-ES epochs, so the final FIC parameter estimates were significantly worse than Euler CMA-ES.

The astute reader might also observe that Euler integration was used to select the best FIC parameters in the validation set. Using DELSSOME in both training and validation sets led to slightly worse (but probably not statistically worse) FIC model parameters than Euler CMA-ES (results not shown). Therefore, we made the decision to suffer a run time penalty by using Euler integration in the validation. Consequently, the run time acceleration decreased from 2000× to 50×. Future improvements in DELSSOME modeling might further speed up FIC parameter estimation by enabling the use of DELSSOME in the validation set.

### 3.3 Limitations and future work

The current DELSSOME instantiation seeks to optimize the excitatory-to-excitatory recurrent strength (w_EE_), excitatory-to-inhibitory connection strength (w_EI_) and noise amplitude (σ). To optimize different parameters (e.g., excitatory and inhibitory gains) or a different mean field model (e.g., Deco et al., 2013), the same neural architecture can likely be used, but the new DELSSOME models will have to be retrained from scratch. However, we note that this is a fundamental limitation for any deep learning approach not limited to the DELSSOME framework.

Finally, if the biophysical model is of a completely different type, e.g., neural field model instead of neural mass model, then the current DNN architecture would need to be modified. However, the key conceptual idea of predicting surrogate statistics directly, rather than generating the actual timeseries would still apply. Similarly, we posit that the modified DNN architecture would require separate model and empirical data embeddings in a latent space prior to their integration and prediction of surrogate statistics (Section 3.1).

### 3.4 Conclusion

In this work, we propose and validate the DELSSOME framework for speeding up the estimation of biophysical model parameters. The massive speedup provided by DELSSOME will enable the large-scale deployment of mechanistic models in population-level neuroscience.

## 4 Methods

### 4.1 Datasets

#### 4.1.1 Human Connectome Project (HCP) dataset

We considered data of 1029 participants from the Human Connectome Project (HCP) S1200 release (Glasser et al., 2013; Van Essen et al., 2013). The participants underwent scanning on a customized Siemens 3T Skyra using a multi-band sequence. Each participant completed four resting-state fMRI (resting-fMRI) runs in two sessions on two different days. The resting-fMRI runs had a repetition time (TR) of 0.72 s at 2 mm isotropic resolution, each lasting 14.4 minutes. Additionally, six runs of diffusion imaging were conducted, each lasting approximately 9 minutes and 50 seconds. Diffusion weighting consisted of 3 shells of b-values (1000, 2000, and 3000 s/mm^2^) with approximately equal number of weighting directions on each shell. Further specifics of the data collection are available elsewhere (Van Essen et al., 2013).

Preprocessing of the HCP fMRI data is detailed in the HCP S1200 manual. We utilized preprocessed (MSMAll) resting-fMRI data, already projected to fsLR surface space, denoised with ICA-FIX, and smoothed by 2 mm. For each run of each participant, the fMRI data were averaged within each Desikan–Killiany region of interest (ROI; Desikan et al., 2006) to create a 68 × 1200 matrix. For each run of each participant, 68 × 68 functional connectivity (FC) matrices were computed by correlating the time courses among all pairs of time courses.

Functional connectivity dynamics (FCD) were computed by defining a 60-second window (equivalent to 83 time points or TRs) and sliding the window frame by frame, resulting in 1118 sliding windows (Zalesky et al., 2014; Hansen et al., 2015; Leonardi & Van De Ville, 2015). FC was computed within each sliding window for each participant’s run. Each sliding window FC matrix was then vectorized by only considering the upper triangular entries. The vectorized FCs were correlated, resulting into a 1118 × 1118 FCD matrix for each run of each participant.

For diffusion MRI, the processing begins with *b*0 intensity normalization, followed by the calculation of susceptibility-induced B_0_ field deviations using *b*0 images acquired in both phase-encoding directions. The full diffusion timeseries from both phase-encoding directions is then processed with the “eddy” tool, which corrects for eddy current distortions and subject motion. Gradient distortion is subsequently corrected, and the *b*0 image is aligned to the T1-weighted image using boundary-based registration (BBR). The corrected diffusion data output from the “eddy” step are resampled into the native structural space at 1.25 mm resolution and masked. Additionally, diffusion directions and gradient deviation estimates are appropriately rotated and registered into the structural space (Glasser et al., 2013). Probabilistic tractography with the Anatomically-Constrained Tractography (ACT) (Smith et al., 2012) was performed on fiber orientation distribution (FOD) images was performed in each participant, using the second-order integration over fiber orientation distribution (iFOD2) algorithm provided by MRtrix3 (Tournier et al., 2010, 2019). 5,000,000 streamlines were sampled to generate tractograms, which were then filtered using Spherical-deconvolution informed filtering of tracks (SIFT2; Smith et al., 2015). This process generated a structural connectivity (SC) matrix for each participant, where each entry represented the weighted sum of the streamlines, approximating the mean fiber density (Smith et al., 2022) between two Desikan-Killiany ROIs.

#### 4.1.2 Philadelphia Neurodevelopment Cohort (PNC) dataset

Neuroimaging data were acquired from a community-based cohort comprising 1601 youth aged 8.1 to 23.1 (mean = 14.94, standard deviation = 3.69, male/female = 764/837) enrolled in the Philadelphia Neurodevelopmental Cohort (PNC). Comprehensive details regarding data collection procedures and participant demographics can be found in previous studies (Satterthwaite et al., 2014; Calkins et al., 2015). A single resting-fMRI scan was collected for each participant. Consistent with our previous work (Zhang et al., 2024a), a total of 885 participants (aged 8.2 to 23.0 at initial visit, mean = 15.66, standard deviation = 3.36) were used for all analyses.

All neuroimaging data were acquired using a Siemens Trio 3T scanner, with detailed descriptions of imaging protocols and acquisition parameters available elsewhere (Satterthwaite et al., 2014; Calkins et al., 2015). In brief, parameters included TR = 3000 ms, TE = 32 ms, flip angle = 90°, FOV = 192 × 192 mm^2^, matrix = 64 × 64, 46 slices, slice thickness/gap = 3/0 mm, effective voxel resolution = 3.0 × 3.0 × 3.0 mm^3^, and a total of 124 volumes.

Preprocessing followed previous studies (Larsen et al., 2022; Zhang et al., 2024a). BOLD runs were slice time–corrected and then motion-corrected. Susceptibility distortion was estimated and used to compute a corrected BOLD reference for more accurate co-registration with the anatomical reference. The BOLD reference was co-registered to the T1w reference using boundary-based registration. Co-registration was configured with nine degrees of freedom to account for distortions remaining in the BOLD reference. Six head motion parameters (corresponding to rotation and translation parameters) were estimated before any spatiotemporal filtering. The motion-correcting transformations and field distortion correcting warp were applied to the BOLD time series in a single step. Finally, the volumetric data was projected to fsLR surface space.

Finally, nuisance regression incorporated anatomical CompCor (aCompCor), with principal components estimated after high-pass filtering of preprocessed BOLD time series (using a discrete cosine filter with a 128-s cutoff). Five CompCor components were extracted from cerebrospinal fluid (CSF) and white matter (WM) masks. In total, 17 regressors were jointly regressed from the BOLD time series, including 6 head motion parameters and their temporal derivatives, and top 5 aCompCor components (Zhang et al., 2024a).

FC and FCD for the PNC datasets were computed in the same manner as the HCP dataset. However, due to a longer TR in the PNC dataset compared to the HCP dataset, the length of each sliding window for FCD matrix computation was set to 20 timepoints (or TRs) to maintain a consistent window length of 60 seconds (Leonardi & Van De Ville, 2015).

### 4.2 DELSSOME neural network architecture

**Figure 6.**
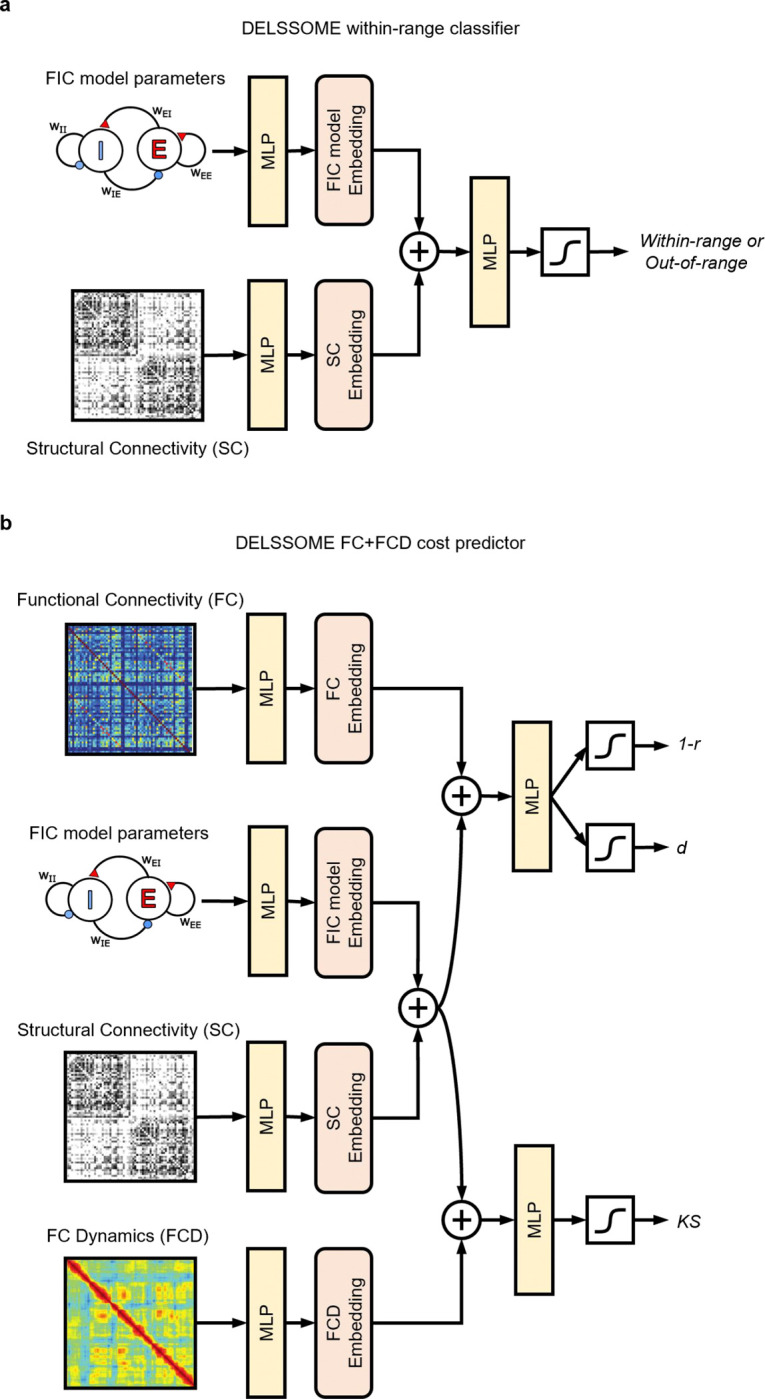
DELSSOME neural network architectures. **a.** Overview of the DELSSOME within-range classifier. The inputs to this model are FIC parameters and structural connectivity (SC). The FIC parameters and SC were passed through separate MLP and then added to generate a vector representing sufficient statistics of the FIC model. This FIC model was passed through another MLP to predict whether the FIC parameters would lead to an excitatory firing rate within the range of 2.7 to 3.3 Hz. **b.** Overview of the DELSSOME FC+FCD cost predictor. The inputs to this model are FIC model parameters, SC, empirical functional connectivity (FC) matrix and empirical FC dynamics (FCD). The FIC parameters and SC were passed through separate MLP and then added to generate a vector representing sufficient statistics of the FIC model. The FC and FCD were also passed through separate MLP to generate FC and FCD embeddings. The FC and FIC model embeddings were added together and passed through another MLP to predict FC costs *1-r* and *d*. The FCD and FIC model embeddings were added together and passed through another MLP to predict FCD cost *KS*.

To avoid computationally intensive numerical integration (Figure 1c) in the CMA-ES evaluation, we proposed the DELSSOME (Deep Learning for Surrogate Statistics Optimization in Mean Field Modeling) within-range classifier to directly predict whether a set of FIC parameters would lead to within-range firing rates (Figure 1d). The inputs to the DELSSOME within-range classifier were the 10 FIC parameters and the structural connectivity (SC) matrix (Figure 6a). Separate multilayer perceptron (MLP) modules were used to embed the FIC parameters and the SC. These embeddings represented surrogate statistics encapsulating essential information from FIC parameters and SC matrix. The FIC parameter embeddings and SC embeddings were then added to result in a final FIC model embedding. The final model embedding was then fed through another MLP to predict whether a given set of FIC parameters and SC would result in a within-range firing rate. More details of the DELSSOME within-range classifier and the hyperparameters are found in Supplementary Methods S5.

The set up of the DELSSOME FC+FCD cost predictor was similar (Figure 6b). The inputs to the DELSSOME FC+FCD cost predictor were the 10 FIC parameters and the structural connectivity (SC) matrix. Separate multilayer perceptron (MLP) modules were used to embed the FIC parameters and the SC. The FIC parameter embeddings and SC embeddings were then added together resulting in a final FIC model embedding.

Since the FC cost involved comparing the simulated FC and empirical FC, so the empirical FC matrix should be another input to the DELSSOME FC+FCD cost predictor. The empirical FC matrix was fed through an MLP, resulting in an FC embedding. The FC embedding was added with the FIC model embedding, and fed through a final MLP to predict the FC costs *1-r* and *d*.

Similarly, since the FCD cost involved comparing the simulated FCD and empirical FCD, so the empirical FCD should also be an input to the DELSSOME FC+FCD cost predictor. The empirical FCD matrix was fed through an MLP, resulting in an FCD embedding. The FCD embedding was added with the FIC model embedding, and fed through a final MLP to predict the FCD cost *KS*. More details of the DELSSOME FC+FCD cost predictor and the hyperparameters are found in Supplementary Methods S6.

### 4.3 Training & evaluation of DELSSOME within-range classifier & cost predictor

**Figure 7.**
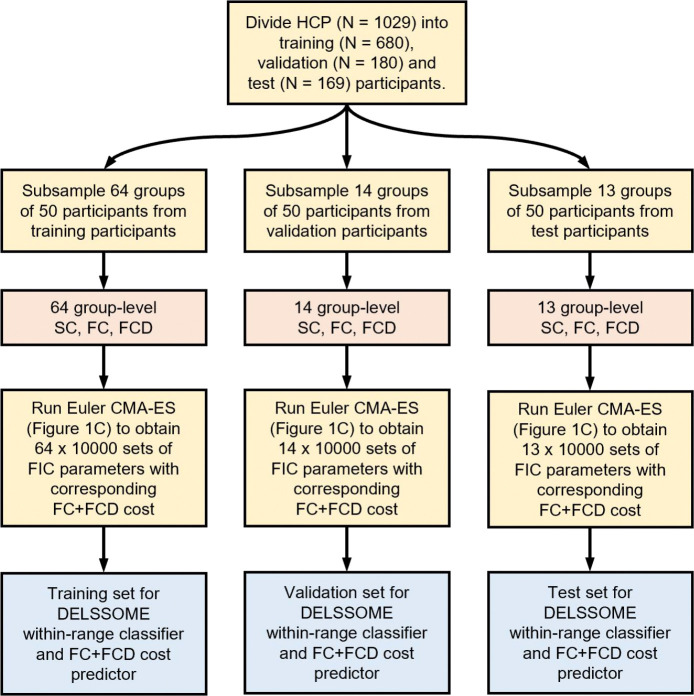
Data generation for training and evaluating DELSSOME within-range firing rate classifier and DELSSOME FC+FCD cost predictor.

To train and evaluate the DELSSOME within-range classifier and the DELSSOME FC+FCD cost predictor, we divided the HCP dataset in training (N = 680), validation (N = 180) and test (N = 169) sets (Figure 7). Within the training set, we repeatedly sampled 50 participants to compute group-average SC, FC and FCD matrices. The sampling was performed 64 times resulting in 64 group-average SC, FC and FCD matrices in the training set. The same procedure was repeated in the validation and test sets separately, yielding 14 group-level SC, FC and FCD for the validation set, as well as 13 SC, FC and FCD for the test set.

For each triplet of group-level SC, FC and FCD matrices, we ran CMA-ES with Euler integration for 100 epochs. Euler integration was performed with time step of 6 ms. Since each CMA-ES epoch employed 100 children, this process generated 10,000 sets of FIC parameters with corresponding ground truth excitatory firing rates and FC+FCD cost, which could be used to train and evaluate the DELSSOME models. Since there were 64, 14 and 13 group-level SC, FC and FCD in the training, validation and test sets respectively, this yielded 640,000, 140,000 and 130,000 training, validation and test samples respectively.

The 640,000 training samples were used to train the DELSSOME within-range classifier, while the 140,000 validation samples were used to tune the hyperparameters. More details of the hyperparameter tuning and final set of hyperparameters are found in Supplementary Method S5. The final trained DELSSOME within-range classifier were then evaluated with the 130,000 test samples.

In the case of the DELSSOME FC+FCD cost predictor, we note that the FIC parameters of 388,413 (out of 640,000) training samples, 87,761 (out of 140,000) validation samples and 79,144 (out of 130,000) test samples resulted in excitatory firing rates within the range of 2.7Hz and 3.3Hz (based on Euler integration). The DELSSOME FC+FCD cost predictor were trained and evaluated only on these “within-range” samples.

### 4.4 Computational cost in training DELSSOME models

Both Euler CMA-ES and DELSSOME CMA-ES are coded in python and run on the same hardware: Intel(R) Xeon(R) Gold 6230 CPU @ 2.10GHz. While the application of trained DELSSOME models is very fast (as reported in the main text), there is significant cost in generating DELSSOME training data and training DELSSOME models.

In the current study, generating DELSSOME training data required 31 hours of compute time with 91 CPU cores, while training the DELSSOME models required 10 hours of compute with using 2 CPU cores. However, we note that this is a one-time cost, while pretrained DELSSOME models can be repeatedly used in different datasets.

### 4.5 Ethics and data availability

Use of de-identified data from the HCP and PNC datasets is approved by the National University of Singapore (NUS) Institutional Review Board (IRB).

The HCP data is publicly available (https://www.humanconnectome.org/). The PNC dataset is publicly available in the Database of Genotypes and Phenotypes (dbGaP accession phs000607.v3.p2).

### 4.6 Code availability

Code for this study, together with pretrained DELSSOME models can be found here (GITHUB_LINK). Co-authors (TZ and FT) reviewed the code before merging into the GitHub repository to reduce the chance of coding errors.

## Supplementary Material

Supplement 1
