# Supplementary material for: Optimizing Biophysical Large-Scale Brain Circuit Models With Deep Neural Networks
Source: bioRxiv. 2025 Apr 7:2025.04.07.647497. Preprint. [Version 1] doi: 10.1101/2025.04.07.647497 (PMC12026898; doi:10.1101/2025.04.07.647497)
Supplement: Supplement 1 [file media-1.docx]

**Optimizing Biophysical Large-Scale Brain Circuit Models**

**With Deep Neural Networks**

# Supplemental Material

This supplemental material consists of Supplemental Methods and Supplemental Results to complement the Methods and Results sections in the main text.

# Supplemental Methods

## S1. Details of the FIC model

The feedback inhibition control (FIC) model (Deco et al., 2014) was derived from the mean field reduction of a spiking neuronal network model (Brunel & Wang, 2001; Wong & Wang, 2006). The detailed derivation of the FIC model can be found in a previous study (Deco et al., 2014). The differential equations governing the $j$-th cortical region are shown below:

$$\begin{aligned} I_{j}^{\left( E \right)}=W_{E}I_{0}+w_{EE}J_{NMDA}S_{j}^{\left( E \right)}+GJ_{NMDA}\sum_{k} C_{jk}S_{k}^{\left( E \right)}-w_{IE}S_{j}^{\left( I \right)}\#\left( 1 \right) \end{aligned}$$

$$\begin{aligned} I_{j}^{\left( I \right)}=W_{I}I_{0}+w_{EI}J_{NMDA}S_{j}^{\left( E \right)}-w_{II}S_{j}^{\left( I \right)} \#\left( 2 \right) \end{aligned}$$

$$\begin{aligned} r_{j}^{\left( E \right)}=\phi\left( I_{j}^{\left( E \right)} \right)=\frac{a_{E}I_{j}^{\left( E \right)}-b_{E}}{1-\exp\left( -d_{E}\left( a_{E}I_{j}^{\left( E \right)}-b_{E} \right) \right)} \#\left( 3 \right) \end{aligned}$$

$$\begin{aligned} r_{j}^{\left( I \right)}=\phi\left( I_{j}^{\left( I \right)} \right)=\frac{a_{I}I_{j}^{\left( I \right)}-b_{I}}{1-\exp\left( -d_{I}\left( a_{I}I_{j}^{\left( I \right)}-b_{I} \right) \right)} \#\left( 4 \right) \end{aligned}$$

$$\begin{aligned} \frac{dS_{j}^{\left( E \right)}}{dt}=-\frac{S_{j}^{\left( E \right)}}{\tau_{E}}+\left( 1-S_{j}^{\left( E \right)} \right)\gamma r_{j}^{\left( E \right)}+\sigma\nu_{j}\left( t \right) \#\left( 5 \right) \end{aligned}$$

$$\begin{aligned} \frac{dS_{j}^{\left( I \right)}}{dt}=-\frac{S_{j}^{\left( I \right)}}{\tau_{I}}+r_{j}^{\left( I \right)}+\sigma\nu_{j}\left( t \right) \#\left( 6 \right) \end{aligned}$$

where $S$, $r$, and $I$ denote synaptic gating variable, firing rate, and synaptic current respectively. The superscripts $E$ and $I$ represent the excitatory and inhibitory neuronal populations respectively.

The input current $I_{j}^{(E)}$ of the excitatory population of the $j$-th cortical ROI is the sum of four inputs (Equation 1). The first input is the external input current $W_{E}I_{0}$, which might include subcortical delays. The second input is the intra-regional excitatory-to-excitatory current governed by the excitatory-to-excitatory recurrent connection strength $w_{EE}$ scaled by the synaptic coupling constant $J_{NMDA}$. The third input is the inter-regional input, which is controlled by the SC matrix ($C_{jk}$ is the connectivity between regions $j$ and $k$) and scaled by the global constant $G$. The fourth input is the intra-regional negative feedback from the inhibitory population governed by the inhibitory-to-excitatory connection strength $w_{IE}$.

The input current $I_{j}^{(I)}$ of the inhibitory population of the $j$-th cortical ROI is the sum of three inputs (Equation 2). The first input is the external input current $W_{I}I_{0}$. The second input is the intra-regional positive feedback from the excitatory population governed by the excitatory-to-inhibitory connection strength *w_EI_* scaled by the synaptic coupling constant $J_{NMDA}$. The third input is the intra-regional inhibitory-to-inhibitory current governed by the inhibitory-to-inhibitory recurrent connection strength $w_{II}$.

The excitatory input current $I_{j}^{(E)}$ and inhibitory input current $I_{j}^{(I)}$ are transformed into firing rates via the input-output functions specified in Equations 3 and 4. Following previous studies (Deco et al., 2014), the parameters of the input-output function were set to be excitatory gain $a_{E}=310$n/C, inhibitory gain $a_{I}=615$n/C, $b_{E}=125$Hz, $b_{I}=177$Hz, $d_{E}=0.16$s and $d_{I}=0.087$s. Finally, the rate of change of the synaptic gating variables $S_{j}^{(E)}$ and $S_{j}^{(I)}$ are computed via equations 5 and 6. Following previous studies (Deco et al., 2014), the kinetic parameters for synaptic activities $\tau_{E}$, $\tau_{I}$and $\gamma$ were set to 100ms, 10ms and 0.641 respectively. $\nu_{j}\left( t \right)$ corresponds to uncorrelated standard Gaussian noise with the noise amplitude being controlled by $\sigma$.

Following the original study (Deco et al., 2014), $w_{II}$, $W_{E}$, $W_{I}$, $I_{0}$ and $J_{NMDA}$ were set to 1, 1, 0.7 0.382nA and 0.15nA respectively in the current study. The inhibitory-to-excitatory connection strength $w_{IE}$was computed analytically to ensure that the excitatory firing rate is maintained to be around 3Hz (De Kock & Sakmann, 2008). We note that this analytical computation assumes a noiseless system, so in practice, we imposed a constraint that the firing rate is between 2.7Hz to 3.3Hz. During the estimation of the FIC model (next section), parameters were rejected if firing rates fall outside this range.

The local circuit parameters, including $w_{EE}$ (excitatory-to-excitatory recurrent connection strength), $w_{EI}$ (excitatory-to-inhibitory connection strength), $\sigma$ (noise amplitude) and $G$ (global SC scaling constant) are unspecified and will be inferred by fitting to empirical fMRI data. Given a fixed set of model parameters, equations 1 to 6 were used to simulate the time courses of excitatory and inhibitory synaptic gating variables ($S_{j}^{(E)}$ and $S_{j}^{(I)}$) of each ROI with a fixed local circuit parameter set. The regional E/I ratio was defined as the average temporal ratio between $S_{j}^{(E)}$ and $S_{j}^{(I)}$. The mean cortical E/I ratio was then derived from averaging regional E/I ratios across all cortical ROIs. Additionally, the simulated excitatory synaptic gating variables ($S_{j}^{(E)}$) were fed into the Balloon-Windkessel hemodynamic model to generate fMRI BOLD signals (Stephan et al., 2007; Deco et al., 2014). The simulated fMRI BOLD signals were utilized to generate simulated static FC and FCD.

## S2. Optimizing the FIC model with CMA-ES in the HCP dataset

As mentioned in the previous section, we seek to optimize $w_{EE}$ (excitatory-to-excitatory recurrent connection strength), $w_{EI}$ (excitatory-to-inhibitory connection strength), $\sigma$ (noise amplitude) and $G$ (global SC scaling constant). We assumed that $w_{EE}$, $w_{EI}$ and $\sigma$ were spatially heterogeneous. Assuming a 68-region Desikan-Killiany parcellation (Desikan et al., 2006), this resulted in a different $w_{EE}$, $w_{EI}$ and $\sigma$ for each brain region, yielding 68 × 3 + 1 = 205 parameters to be estimated.

To further constrain the parameter space (Zhang et al., 2024), w_EE_​, w_EI_​, and $\sigma$ were parameterized as linear combinations of the principal resting-state functional connectivity gradient (Margulies et al., 2016) and the T1w/T2w myelin map (Glasser & Van Essen, 2011).

$$\begin{aligned} w_{EE,j}=a+b\times{T1w/T2w \mathrm{ratio}}_{j}+c\times{FC gradient}_{j} \#\left( 7 \right) \end{aligned}$$

$$\begin{aligned} w_{EI,j} =d+e\times{T1w/T2w \mathrm{ratio}}_{j}+f\times{FC gradient}_{j} \#\left( 8 \right) \end{aligned}$$

$$\begin{aligned} \sigma_{j}=g+h\times{T1w/T2w \mathrm{ratio}}_{j}+i\times{FC gradient}_{j}\#\left( 9 \right) \end{aligned}$$

where *j* denotes the *j*-th region. By employing this parameterization strategy, the number of “free” numbers was reduced to 3 × 3 + 1 = 10 parameters (including $G$).

The 10 parameters were optimized using CMA-ES by minimizing dissimilarity of the simulated FC and FC to empirical FC and FCD. The agreement between empirical and simulated FC matrices was defined as the Pearson’s correlation (*r*) between the z-transformed upper triangular entries of the two matrices. Larger *r* indicates more similar static FC. However, Pearson’s correlation does not account for scale difference, so we also computed the absolute difference (*d*) between the means of the empirical and simulated FC matrices (Demirtaş et al., 2019). A smaller d indicates more similar static FC.

We note that there is no temporal correspondence between simulated and empirical FCD matrices, so we cannot simply use the Euclidean distance to measure dissimilarity. Instead, the dissimilarity between simulated and empirical FCD matrices was quantified by using the Kolmogorov-Smirnov (*KS*) distance. Here, the KS distance was defined as the maximum distance between the cumulative distribution functions (CDFs) constructed by collapsing the upper triangular entries of simulated and empirical FCD matrices (Hansen et al., 2015; Kong et al., 2021). Hence, a small *KS* distance indicated 2 similar CDFs, therefore 2 similar FCD matrices. Because the *KS* distance was computed by collapsing the upper triangular entries of the FCD matrices, no temporal correspondence was assumed.

The overall FC+FCD cost was defined as (1 – *r*) + *d* + *KS*. A smaller cost indicates better agreement between simulated and empirical fMRI. Throughout the study, we note that FC+FCD cost was computed at the group level. For more details about how group-level FC, FCD and SC are computed, see Supplementary Methods S3.

In the case of evaluating DELSSOME CMA-ES in the HCP dataset (Figure 3), recall that the HCP test participants (N = 169) were further divided into the FIC model inversion training (N = 57), validation (N = 56) and test (N = 56) sets (Figure 3a). Group-average FC and FCD were computed separately in the training, validation and test sets. Group-average SC, FC gradient and T1w/T2w ratio was computed only in the HCP training set (N = 680) and were used throughout the analysis.

Euler CMA-ES was run on the FIC model inversion training set for 100 epochs. FC+FCD cost was computed using FIC model inversion training set FC and FCD. The best candidate parameter set from each epoch was selected, yielding 100 candidate parameter sets. The 100 candidate parameter sets were then evaluated in the FIC model inversion validation set using the FIC model inversion validation set FC and FCD. Finally, the top parameter set from the validation set was evaluated in the FIC model inversion test set using the FIC model inversion test set FC and FCD.

When computing FC+FCD cost using Euler integration, the FIC model was simulated three times, and the average simulated FC and FCD were used to compute the FC+FCD cost. Euler integration in the FIC model inversion training set was performed with timestep of 6ms, while Euler integration in the FIC model inversion validation and test sets was performed with timestep of 0.5ms.

DELSSOME CMA-ES utilized the same procedure as Euler CMA-ES, except that we used DELSSOME instead of Euler integration in the FIC model inversion training set. However, we still used Euler integration in the FIC model inversion validation and test sets.

## S3. Group-level FC, FCD and SC

Across different sections of this study, group-average FC, FCD and SC were computed based on different groups of participants. Our procedure followed that of our previous studies (Kong et al., 2021; Zhang et al., 2024). More specifically, to compute group-level FC across a group of participants, we applied Fisher-r-to-z transformation to the FC matrix of each run of each participant. The FC matrices were then averaged across runs within each participant, followed by averaging across all participants and then applying the inverse Fisher-r-to-z transformation.

In the case of FCD, recall that the FCD each participant was a W × W matrix, where W is the number of sliding windows (W = 1118 in HCP and 109 in PNC). Unlike static FC, FCD matrices could not be directly averaged across participants due to the lack of temporal correspondence during resting-state. As discussed in the previous section, we used the Kolmogorov–Smirnov (KS) distance to measure the distance between the two FCD matrices, consistent with previous studies (Zhang et al., 2024). The KS distance between two FCD matrices was defined as the maximum distance between the cumulative distribution functions (CDFs) obtained by collapsing the upper triangular entries of simulated and empirical FCD matrices, so no temporal correspondence was assumed. Therefore, given the FCD matrices of a group of participants, instead of averaging the FCD matrices, we averaged the CDF of the FCD matrices.

To generate a group-level SC matrix, a thresholding procedure was first applied to remove false positives (de Reus & van den Heuvel, 2013). More specifically, if <50% of participants had a non-zero value in a particular entry in the SC matrix, then the entry is set to zero in all individual-level SC matrices. Then averaging across participants with non-zero streamlines, log-transforming the averaged values, and setting the main diagonal entries to zero were performed. Group-level SCs were computed separately for a particular group of participants, with the maximum value normalized to 0.02.

## S4. Optimizing the FIC model with CMA-ES in the PNC dataset

To evaluate whether DELSSOME CMA-ES generalizes to a new dataset, the DELSSOME models trained from the HCP dataset were applied directly to the Philadelphia Neurodevelopment Cohort (PNC) dataset (Figures 4 and 5). Because there was no diffusion data and T1w/T2w ratio in the PNC dataset, group-average SC, FC gradient and T1w/T2w ratio from the HCP training set (N = 680) were used.

In the case of associating E/I ratio with age, recall that PNC participants were sorted according to age and divided into 29 age groups (with 30 or 31 participants in each group). Within each age group, 15 participants were randomly selected as the validation set, while the remaining participants were assigned to the training set. Group-average FC and FCD were computed separately in the training and validation sets.

For each age group, DELSSOME CMA-ES was applied to the training set for 50 epochs with FC+FCD cost computed using the training set FC and FCD. The procedure was repeated five times with different random initializations, resulting in 250 candidate parameter sets. The 250 parameter sets were evaluated in the validation set with Euler integration using the validation set FC and FCD. The best candidate parameter set was then used to generate an excitation/inhibition (E/I) ratio map using Euler integration.

When computing FC+FCD cost using Euler integration, the FIC model was simulated three times, and the average simulated FC and FCD were used to compute the FC+FCD cost. When computing E/I ratio, the FIC model was also simulated three times, and the E/I ratio estimates were averaged across the three simulations. Euler integration in the training set and validation set was performed with timestep of 6ms, while Euler integration in the computation of the final E/I ratio was performed with timestep of 0.5ms.

Furthermore, consistent with our previous study (Zhang et al., 2024), during CMA-ES, we additionally imposed the constraints that the spatial correlation between w_EI_ and T1w/T2w ratio should be negative, while the spatial correlation between w_EI_ and FC gradient should be positive. Euler CMA-ES followed the same procedure as DELSSOME CMA-ES, except that Euler integration was used throughout.

In the case of associating E/I ratio with cognitive performance, the same DELSSOME CMA-ES and Euler CMA-ES procedures were used as the age analysis, except that instead of participants divided into different age groups, we now have participants divided into different low and higher performance groups.

## S5. DELSSOME within-range classifier

As mentioned in the main text, the inputs to the DELSSOME within-range classifier were the 10 FIC parameters and the structural connectivity (SC) matrix (Figure 6a). The model was trained on the HCP training set (N = 640,000), and hyperparameters were empirically determined on the HCP validation set (N = 140,000). We emphasized that the HCP test set (N = 130,000) was not used to train or tune the hyperparameters.

In the case of the 68-region Desikan-Killiany parcellation, equations 7 to 9 are used to “expand” 9 FIC parameters into 68 × 3 = 204 parameters. Together with the global *G* parameter, there were 205 parameters, which were fed into a two-layer multilayer perceptron (MLP) module to embed the FIC parameters. The first layer comprised 88 nodes, while the output layer contained 111 nodes. ReLU activation and BatchNorm were utilized for each layer.

The upper triangle entries of the 68 × 68 SC matrix (2278 entries) were vectorized and also fed into a separate two-layer MLP module to embed the SC. The first layer comprised 984 nodes, while the output layer contained 111 nodes. ReLU activation and BatchNorm were utilized for each layer.

The FIC and SC embeddings represented surrogate statistics encapsulating essential information from FIC parameters and SC matrix. The FIC parameter embeddings and SC embeddings were then added to result in a final FIC model embedding vector of length 111.

The final model embedding was then fed through another MLP to predict the probability that a given set of FIC parameters and SC would result in a within-range firing rate (between 2.7Hz and 3.3Hz). In this case, based on the validation set, we found that the MLP did not need any hidden layer, so the MLP is simply a linear combination of the model embedding vector (of length 111) followed by the sigmoid function to output the probability of being within-range. For the actual prediction, the probability was thresholded at 50%.

For the loss function, we used the Binary Cross Entropy (BCE). During training, we used the Adam optimizer (Kingma, 2014) with learning rate of 5e-4, accompanied by an exponential learning rate scheduler with reduction rate gamma=0.98. The training was performed for 30 epochs with batch size of 256. After training, we picked the model from the epoch where the DELSSOME classifier exhibited the smallest validation BCE loss.

In the case of the 100-region Yan parcellation, equations 7 to 9 are used to “expand” 9 FIC parameters into 100 × 3 = 300 parameters. Together with the global *G* parameter, there were 301 parameters, which were fed into a two-layer multilayer perceptron (MLP) module to embed the FIC parameters. The first layer comprised 220 nodes, while the output layer contained 173 nodes. ReLU activation and BatchNorm were utilized for each layer.

The upper triangle entries of the 100 × 100 SC matrix (4950 entries) were vectorized and also fed into a separate two-layer MLP module to embed the SC. The first layer comprised 3985 nodes, while the output layer contained 173 nodes. ReLU activation and BatchNorm were utilized for each layer.

The FIC parameter embeddings and SC embeddings were then added to result in a final FIC model embedding of length 173. The final model embedding was then fed through another MLP to predict the probability that a given set of FIC parameters and SC would result in a within-range firing rate (between 2.7Hz and 3.3Hz). In this case, based on the validation set, we found that the MLP did not need any hidden layer, so the MLP is simply a linear combination of the model embedding vector (of length 173) followed by the sigmoid function to output the probability of being within-range. For the actual prediction, the probability was thresholded at 50%.

For the loss function, we used the Binary Cross Entropy (BCE). During training, we used the Adam optimizer (Kingma, 2014) with learning rate of 3e-4, accompanied by an exponential learning rate scheduler with reduction rate gamma=0.98. The training was performed for 30 epochs with batch size of 256. After training, we picked the model from the epoch where the DELSSOME classifier exhibited the smallest validation BCE loss.

## S6. DELSSOME FC+FCD cost predictor

As mentioned in the main text, the inputs to the DELSSOME FC+FCD cost predictor were the 10 FIC parameters and the structural connectivity (SC) matrix, as well as the empirical FC and FCD matrices (Figure 6b). The model was trained on the HCP training set (N = 640,000), and hyperparameters were empirically determined on the HCP validation set (N = 140,000). We emphasized that the HCP test set (N = 130,000) was not used to train or tune the hyperparameters.

In the case of the 68-region Desikan-Killiany parcellation, equations 7 to 9 are used to “expand” 9 FIC parameters into 68 × 3 = 204 parameters. Together with the global *G* parameter, there were 205 parameters, which were fed into a two-layer multilayer perceptron (MLP) module to embed the FIC parameters. The first layer comprised 88 nodes, while the output layer contained 111 nodes. ReLU activation and BatchNorm were utilized for each layer.

The upper triangle entries of the 68 × 68 SC matrix (2278 entries) were vectorized and also fed into a separate two-layer MLP module to embed the SC. The first layer comprised 984 nodes, while the output layer contained 111 nodes. ReLU activation and BatchNorm were utilized for each layer.

The FIC and SC embeddings represented surrogate statistics encapsulating essential information from FIC parameters and SC matrix. The FIC parameter embeddings and SC embeddings were then added to result in a final FIC model embedding vector of length 111.

Since the FC cost involved comparing the simulated FC and empirical FC, so the empirical FC matrix was another input to the DELSSOME FC+FCD cost predictor. The upper triangle entries of the 68 × 68 FC matrix (2278 entries) were vectorized and also fed into a separate three-layer MLP module to embed the FC. The first layer comprised 313 nodes, while the second layer contained 899 nodes. The output layer contains 111 nodes. ReLU activation and BatchNorm were utilized for the second and the third layer.

The resulting FC embedding vector (of length 111) and the FIC model embedding vector (of length 111) were added together and passed through separate MLP to predict the FC costs *1-r* and *d* respectively. Based on the validation set, we found that the MLPs did not need any hidden layer. Therefore, each MLP is simply a linear combination of the input vector (of length 111) followed by the sigmoid function to predict *1-r* (or *d*).

Similarly, the FCD cost involved comparing the simulated FCD and empirical FCD, so the empirical FCD was another input to the DELSSOME FC+FCD cost predictor. The empirical FCD was represented as a probability distribution function (pdf) of FCD values (which ranged from -1 to 1). The range was discretized into 10,000 equal sized bins, so the FCD was essentially a vector of length 10,000 that summed to one. The FCD vector was fed into a three-layer MLP module to embed the FCD. The first layer comprised 1334 nodes, while the second layer contained 5835 nodes. The output layer contains 111 nodes. ReLU activation and BatchNorm were utilized for the second and the third layer.

The resulting FCD embedding vector (of length 111) and the FIC model embedding vector (of length 111) were added together and passed through another MLP to predict the FCD cost *KS*. Based on the validation set, we found that the MLP did not need any hidden layer, so the MLP is simply a linear combination of the input vector (of length 111) followed by the sigmoid function to predict *KS*.

For the lost function, for each of the three terms in the FC+FCD cost (i.e., *1-r*, *d*, *KS*), we computed the square difference between the predicted cost and the actual cost. The overall cost was then the mean of the three square errors, i.e., mean square error (MSE). During training, we adopted Adam optimizer with learning rate of 5e-3, accompanied by a step learning rate scheduler with step size 3 and reduction rate gamma 0.25. We trained the DELSSOME FC+FCD cost predictor for 15 epochs with batch size 256. After training, we picked the model from the epoch where the DELSSOME FC+FCD cost predictor exhibited the smallest validation MSE.

In the case of the 100-region Yan parcellation, equations 7 to 9 are used to “expand” 9 FIC parameters into 100 × 3 = 300 parameters. Together with the global *G* parameter, there were 301 parameters, which were fed into a two-layer multilayer perceptron (MLP) module to embed the FIC parameters. The first layer comprised 220 nodes, while the output layer contained 173 nodes. ReLU activation and BatchNorm were utilized for each layer.

The upper triangle entries of the 100 × 100 SC matrix (4950 entries) were vectorized and also fed into a separate two-layer MLP module to embed the SC. The first layer comprised 3985 nodes, while the output layer contained 173 nodes. ReLU activation and BatchNorm were utilized for each layer.

The FIC and SC embeddings represented surrogate statistics encapsulating essential information from FIC parameters and SC matrix. The FIC parameter embeddings and SC embeddings were then added to result in a final FIC model embedding vector of length 173.

Since the FC cost involved comparing the simulated FC and empirical FC, so the empirical FC matrix was another input to the DELSSOME FC+FCD cost predictor. The upper triangle entries of the 100 × 100 FC matrix (4950 entries) were vectorized and also fed into a separate three-layer MLP module to embed the FC. The first layer comprised 689 nodes, while the second layer contained 2885 nodes. The output layer contains 173 nodes. ReLU activation and BatchNorm were utilized for the second and the third layer.

The resulting FC embedding vector (of length 173) and the FIC model embedding vector (of length 173) were added together and passed through separate MLP to predict the FC costs *1-r* and *d* respectively. Based on the validation set, we found that the MLPs did not need any hidden layer. Therefore, each MLP is simply a linear combination of the input vector (of length 173) followed by the sigmoid function to predict *1-r* (or *d*).

Similarly, the FCD cost involved comparing the simulated FCD and empirical FCD, so the empirical FCD was another input to the DELSSOME FC+FCD cost predictor. The empirical FCD was represented as a probability distribution function (pdf) of FCD values (which ranged from -1 to 1). The range was discretized into 10,000 equal sized bins, so the FCD was essentially a vector of length 10,000 that summed to one. The FCD vector was fed into a three-layer MLP module to embed the FCD. The first layer comprised 688 nodes, while the second layer contained 2553 nodes. The output layer contains 173 nodes. ReLU activation and BatchNorm were utilized for the second and the third layer.

The resulting FCD embedding vector (of length 173) and the FIC model embedding vector (of length 173) were added together and passed through another MLP to predict the FCD cost *KS*. Based on the validation set, we found that the MLP did not need any hidden layer, so the MLP is simply a linear combination of the input vector (of length 173) followed by the sigmoid function to predict *KS*.

For the lost function, for each of the three terms in the FC+FCD cost (i.e., *1-r*, *d*, *KS*), we computed the square difference between the predicted cost and the actual cost. The overall cost was then the mean of the three square errors, i.e., mean square error (MSE). During training, we adopted Adam optimizer with learning rate of 5e-4, accompanied by an exponential learning rate scheduler with reduction rate gamma=0.98. We trained the DELSSOME FC+FCD cost predictor for 15 epochs with batch size 256. After training, we picked the model from the epoch where the DELSSOME FC+FCD cost predictor exhibited the smallest validation MSE.

# Supplemental Results


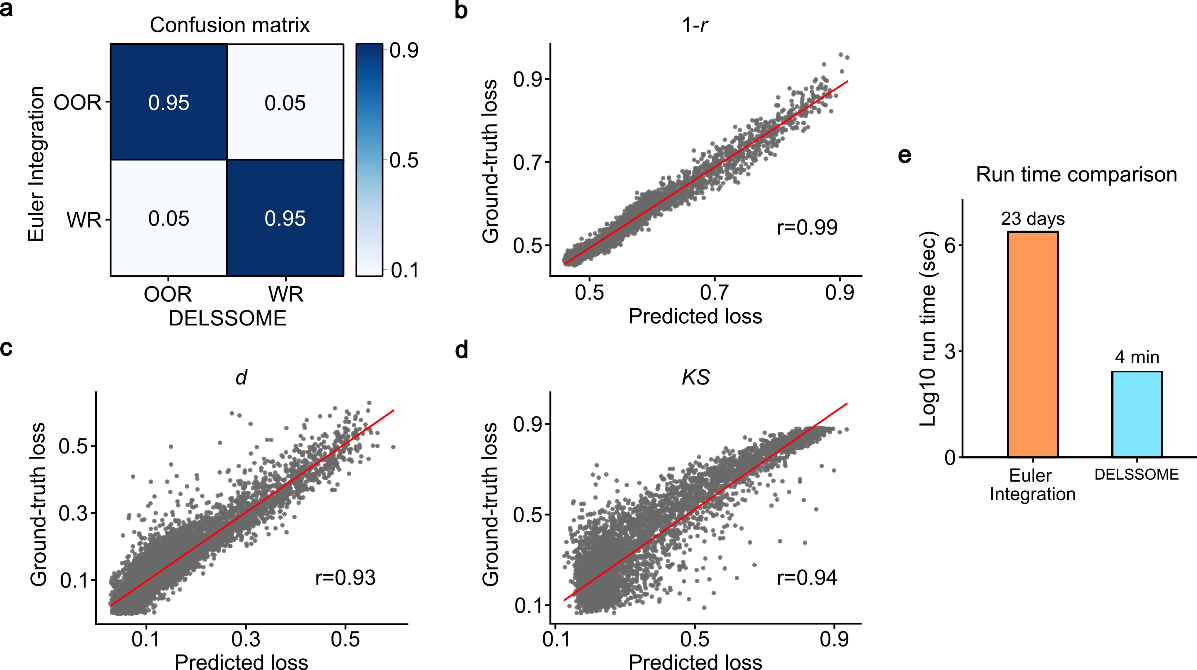


**Figure S1. Test performance of DELSSOME within-range classifier and DELSSOME FC+FCD cost predictor based on the 100-region Yan parcellation.** This figure is the same as Figure 2, but uses the 100-region Yan parcellation (Yan et al., 2023) instead of the Desikan-Killiany parcellation (Desikan et al., 2006). **a.** Test performance (confusion matrix) of DELSSOME within-range classifier. Overall accuracy was 95% (chance accuracy is 68%). OOR: out of range. WR: within range. **b.** Test performance of DELSSOME prediction of static FC cost (*1 - r*). **c.** Test performance of DELSSOME prediction of static FC cost (*d*). **d.** Test performance of DELSSOME prediction of FCD cost (*KS*). The correlation between the predicted and ground truth loss were at least 0.93. In all the analyses, ground truth was defined based on Euler integration, while the DELSSOME models avoided the Euler integration. **e.** Run time (log scale) of DELSSOME versus Euler integration in evaluating FIC model realism. DELSSOME offers a 2000× speed up over Euler integration.


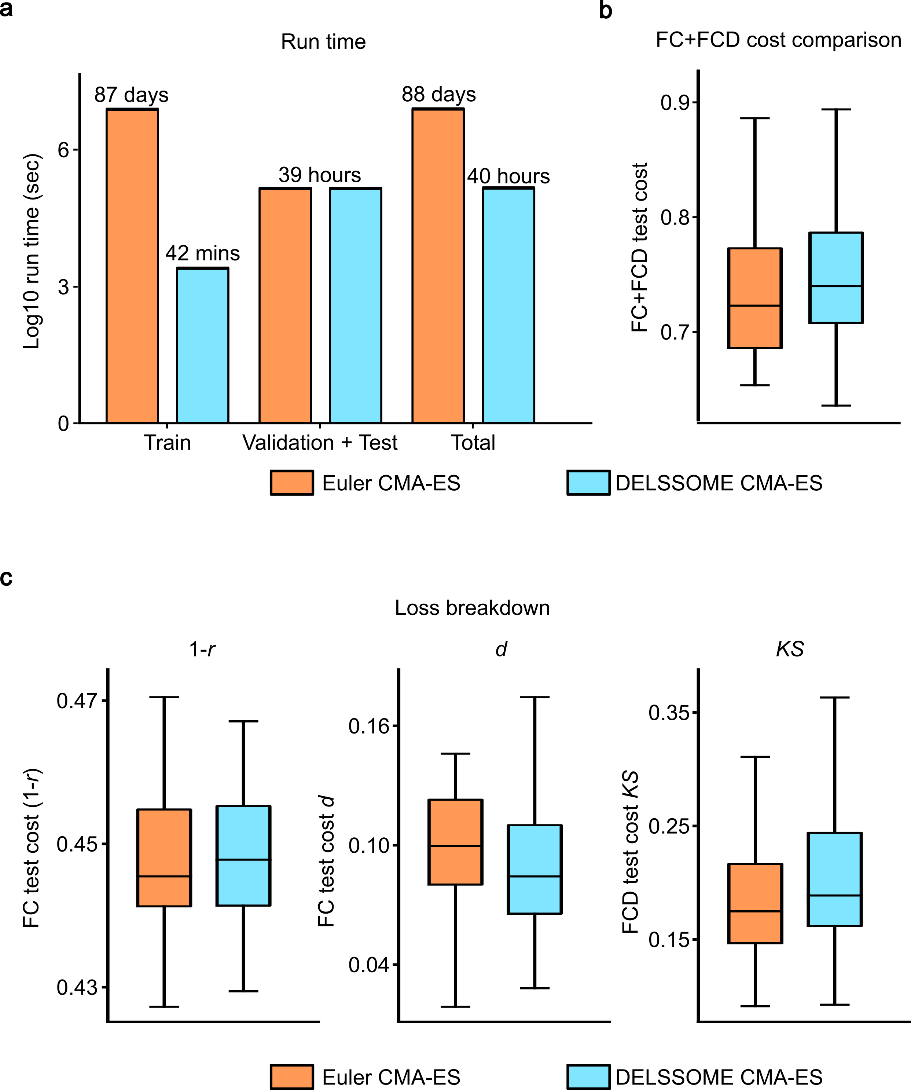


**Figure S2. Comparison of Euler CMA-ES and DELSSOME CMA-ES in the HCP dataset using the 100-region Yan parcellation.** This figure is similar to Figure 3 but uses the Yan parcellation (Yan et al., 2023). **a.** Run time (log scale) of DELSSOME CMA-ES versus Euler CMA-ES. DELSSOME CMA-ES offers a 3000× speed up over Euler CMA-ES in the training phase. If we also included validation and test phases in the run time, DELSSOME CMA-ES offers a 50× speed up over Euler CMA-ES. **b.** Total FC+FCD test cost comparison between DELSSOME CMA-ES and Euler CMA-ES. Each boxplot contains 50 data points corresponding to the 50 repetitions of the procedure in panel (A). **c.** Breakdown of the FC+FCD test cost from panel C into the two FC costs (*1-r* and *d*) and one FCD cost (*KS*). DELSSOME significantly sped up the estimation of the FIC model parameters without any degradation in estimation quality.


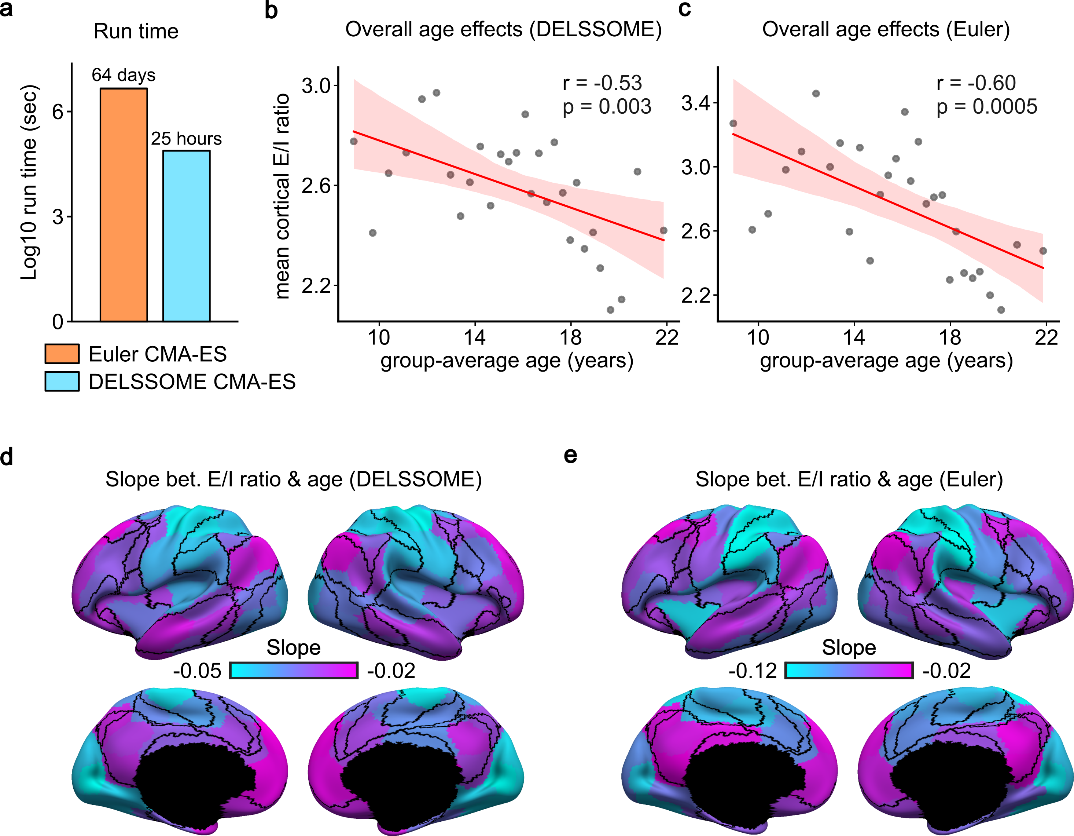


**Figure S3. DELSSOME CMA-ES generalizes to the Philadephia Neurodevelopmental Cohort (PNC) based on the 100-region Yan parcellation without further tuning. a.** Run time comparison between DELSSOME CMA-ES and Euler CMA-ES. DELSSOME CMA-ES offers a 50× speed-up over Euler CMA-ES. **b.** Correlation between age and mean cortical E/I ratio estimated by DELSSOME CMA-ES. **c.** Correlation between age and mean cortical E/I ratio estimated by Euler CMA-ES. **d.** Regression slope between age and regional E/I ratio estimated by DELSSOME CMA-ES. **e.** Regression slope between age and regional E/I ratio estimated by Euler CMA-ES. All slopes in panels d and e are negative. 100 out of 100 regions in panel d and 98 out of 100 regions in panel e were significant after multiple comparisons correction with false discovery rate (FDR) q < 0.05.


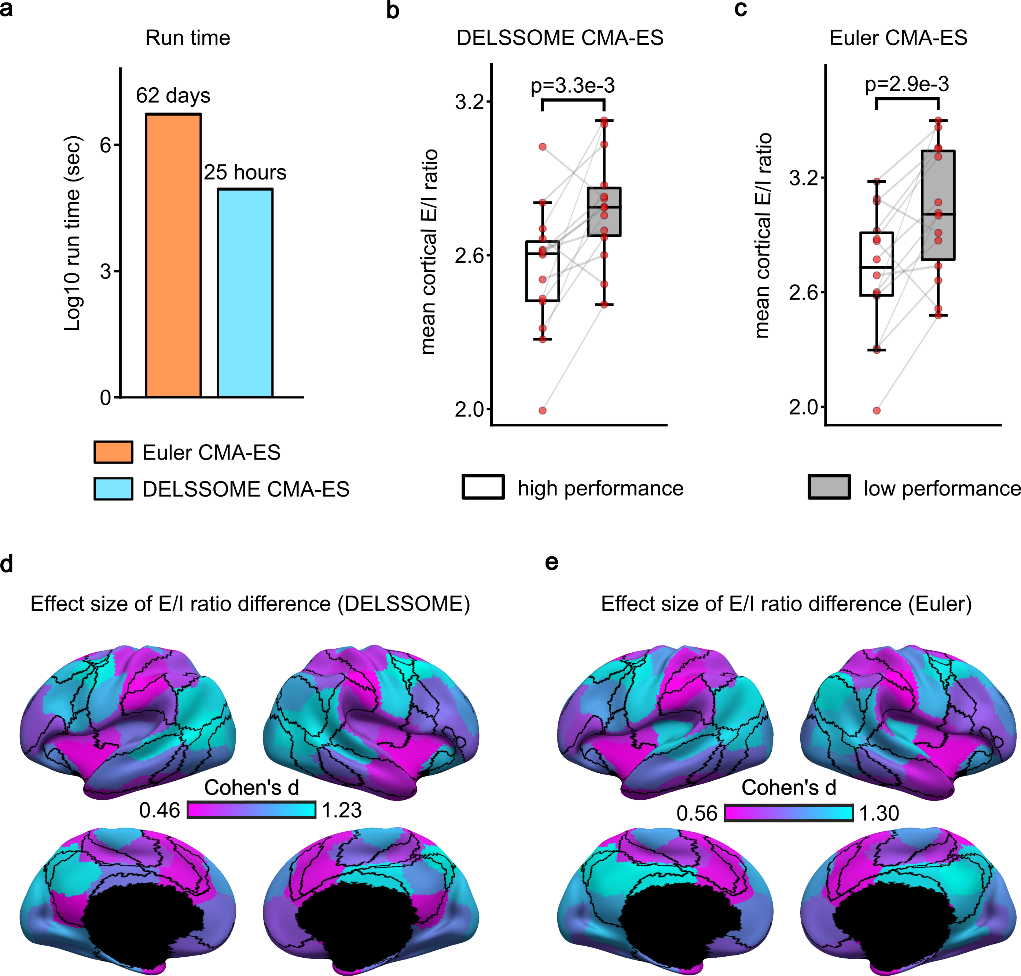


**Figure S4. DELSSOME CMA-ES reveals lower E/I ratio in youth with high cognitive performance, consistent with Euler CMA-ES, based on the 100-region Yan parcellation. a.** Run time comparison between DELSSOME CMA-ES and Euler CMA-ES. DELSSOME CMA-ES offers a 50× speed-up over Euler CMA-ES. **b.** Comparison of mean cortical E/I ratio between high-performance and low-performance groups estimated by DELSSOME CMA-ES. **c.** Comparison of mean cortical E/I ratio between high-performance and low-performance groups estimated by Euler CMA-ES. **d.** Regional differences in cortical E/I ratio between high-performance and low-performance groups estimated by DELSSOME CMA-ES. **e.** Regional differences in cortical E/I ratio between high-performance and low-performance groups estimated by Euler CMA-ES. 89 out of 100 regions in panel **d,** while 96 out of 100 regions in panel **e** were significant after FDR correction with q < 0.05.

# Reference

Brunel, N., & Wang, X.-J. (2001). Effects of Neuromodulation in a Cortical Network Model Brunel, N., & Wang, X.-J. (2001). Effects of Neuromodulation in a Cortical Network Model of Object Working Memory Dominated by Recurrent Inhibition. *Journal of Computational Neuroscience*, *11*(1), 63–85. https://doi.org/10.1023/A:1011204814320

De Kock, C. P. J., & Sakmann, B. (2008). High frequency action potential bursts (≥ 100 Hz) in L2/3 and L5B thick tufted neurons in anaesthetized and awake rat primary somatosensory cortex. *The Journal of Physiology*, *586*(14), 3353–3364. https://doi.org/10.1113/jphysiol.2008.155580

de Reus, M. A., & van den Heuvel, M. P. (2013). Estimating false positives and negatives in brain networks. *Neuroimage*, *70*, 402–409.

Deco, G., Ponce-Alvarez, A., Hagmann, P., Romani, G. L., Mantini, D., & Corbetta, M. (2014). How Local Excitation-Inhibition Ratio Impacts the Whole Brain Dynamics. *Journal of Neuroscience*, *34*(23), 7886–7898. https://doi.org/10.1523/JNEUROSCI.5068-13.2014

Demirtaş, M., Burt, J. B., Helmer, M., Ji, J. L., Adkinson, B. D., Glasser, M. F., Van Essen, D. C., Sotiropoulos, S. N., Anticevic, A., & Murray, J. D. (2019). Hierarchical heterogeneity across human cortex shapes large-scale neural dynamics. *Neuron*, *101*(6), 1181–1194.

Desikan, R. S., Ségonne, F., Fischl, B., Quinn, B. T., Dickerson, B. C., Blacker, D., Buckner, R. L., Dale, A. M., Maguire, R. P., & Hyman, B. T. (2006). An automated labeling system for subdividing the human cerebral cortex on MRI scans into gyral based regions of interest. *Neuroimage*, *31*(3), 968–980.

Glasser, M. F., & Van Essen, D. C. (2011). Mapping human cortical areas in vivo based on myelin content as revealed by T1-and T2-weighted MRI. *Journal of Neuroscience*, *31*(32), 11597–11616.

Hansen, E. C., Battaglia, D., Spiegler, A., Deco, G., & Jirsa, V. K. (2015). Functional connectivity dynamics: Modeling the switching behavior of the resting state. *Neuroimage*, *105*, 525–535.

Kingma, D. P. (2014). Adam: A method for stochastic optimization. *arXiv Preprint arXiv:1412.6980*. https://scholar.google.com/scholar?cluster=16194105527543080940&hl=en&inst=569367360547434339&inst=3212728378801010220&oi=scholarr

Kong, X., Kong, R., Orban, C., Wang, P., Zhang, S., Anderson, K., Holmes, A., Murray, J. D., Deco, G., van den Heuvel, M., & Yeo, B. T. T. (2021). Sensory-motor cortices shape functional connectivity dynamics in the human brain. *Nature Communications*, *12*(1), Article 1. https://doi.org/10.1038/s41467-021-26704-y

Margulies, D. S., Ghosh, S. S., Goulas, A., Falkiewicz, M., Huntenburg, J. M., Langs, G., Bezgin, G., Eickhoff, S. B., Castellanos, F. X., Petrides, M., Jefferies, E., & Smallwood, J. (2016). Situating the default-mode network along a principal gradient of macroscale cortical organization. *Proceedings of the National Academy of Sciences*, *113*(44), 12574–12579. https://doi.org/10.1073/pnas.1608282113

Stephan, K. E., Weiskopf, N., Drysdale, P. M., Robinson, P. A., & Friston, K. J. (2007). Comparing hemodynamic models with DCM. *Neuroimage*, *38*(3), 387–401.

Wong, K.-F., & Wang, X.-J. (2006). A recurrent network mechanism of time integration in perceptual decisions. *Journal of Neuroscience*, *26*(4), 1314–1328.

Yan, X., Kong, R., Xue, A., Yang, Q., Orban, C., An, L., Holmes, A. J., Qian, X., Chen, J., & Zuo, X.-N. (2023). Homotopic local-global parcellation of the human cerebral cortex from resting-state functional connectivity. *NeuroImage*, *273*, 120010.

Zhang, S., Larsen, B., Sydnor, V. J., Zeng, T., An, L., Yan, X., Kong, R., Kong, X., Gur, R. C., Gur, R. E., Moore, T. M., Wolf, D. H., Holmes, A. J., Xie, Y., Zhou, J. H., Fortier, M. V., Tan, A. P., Gluckman, P., Chong, Y. S., … Yeo, B. T. T. (2024). In vivo whole-cortex marker of excitation-inhibition ratio indexes cortical maturation and cognitive ability in youth. *Proceedings of the National Academy of Sciences*, *121*(23), e2318641121. https://doi.org/10.1073/pnas.2318641121
